# Genetic Damage to Human Lymphocytes Induced by Contaminated Water in Populations Surrounding Lake Chapala and the Santiago River, Jalisco, México

**DOI:** 10.3390/toxics13100887

**Published:** 2025-10-17

**Authors:** Mónica Reynoso-Silva, Carlos Alvarez-Moya, Fernando Manuel Guzmán-Rubio, Daniela Guadalupe Velázquez-Cruz, Daniel Moreno-Del Río, Blanca Catalina Ramírez-Hernández, Lucía Barrientos-Ramírez, José de Jesús Vargas-Radillo, Paulina Beatriz Gutiérrez-Martínez, Mario Alberto Ruíz-López

**Affiliations:** 1 Environmental Mutagénesis Laboratory, Cellular and Molecular Department, University of Guadalajara, Guadalajara 45200, Mexico; m.reynoso@academicos.udg.mx (M.R.-S.); fernando.guzman4674@alumnos.udg.mx (F.M.G.-R.); daniela.vcruz@alumnos.udg.mx (D.G.V.-C.); daniel.moreno4614@alumnos.udg.mx (D.M.-D.R.); 2 Applied Ecology Department, University of Guadalajara, Guadalajara 45200, Mexico; 3 Department of Wood, Cellulose and Paper, University of Guadalajara, CUCEI, Road Ing Ramón Padilla Sánchez 2100, Las Agujas, Zapopan 44600, Mexico; lucia.barrientos@academicos.udg.mx (L.B.-R.); j.vargas@academicos.udg.mx (J.d.J.V.-R.); 4 Department of Environmental Sciences, University of Guadalajara, Guadalajara 45200, Mexico; paulina.gutierrez@academicos.udg.mx; 5 Department of Botany and Zoology, University of Guadalajara, Nextipac, Zapopan 45510, Mexico; mario.rlopez@academicos.udg.mx

**Keywords:** pollution, DNA damage, genotoxicity, carcinogenicity, comet assay

## Abstract

Polluted water in the Chapala–Santiago basin (CSB) contains several genotoxic substances that present risks to human health, particularly among residents of communities surrounding Lake Chapala and the Santiago River, where a high prevalence of cancer has been documented. For this reason, it is necessary to study the genotoxic activity of these waters and the genetic damage in inhabitants of the surrounding populations. This study assessed the genotoxicity of water in various communities in the CSB and evaluated DNA damage to lymphocytes in residents of nearby locations. The alkaline comet assay was employed to evaluate water genotoxicity and DNA damage to lymphocytes in residents living near these waters. A standardized questionnaire was distributed to participants of this study to evaluate their exposure to polluted water. Significant genotoxic activity (*p* < 0.05) was observed in the lymphocytes of individuals exposed to contaminated water (tail length in Puente Grande 27.88 ± 5.4 compared to 3.77 ± 1.64 of negative control), along with notable DNA damage (*p* ≤ 0.05) to the lymphocytes of residents living in proximity to these waters (tail length in Juanacatlán 12.3 ± 3.4 compared to 1.4 ± 0.74 of negative control). The waters of the CSB possess the capacity to cause DNA damage; meanwhile, genotoxicity increases from Chapala to El Salto due to the additional input of genotoxic contaminants, thereby elevating the cancer risk for the exposed population. The comet test proved to be a useful tool that allowed data to be obtained quickly and reliably.

## 1. Introduction

River pollution has detrimental impacts on nearby rural riparian communities [1]. In Mexico, 75% of the population is concentrated within 13 hydrological basins, with the Chapala–Santiago basin (CSB) housing the largest human population in the country. The Santiago River is distinguished by its significant pollution and the lack of systematic environmental monitoring policies to help with this situation [2,3,4]. Residents of communities near the CSB are directly exposed to water contaminated with polychlorinated biphenyls (PCBs) and polybrominated diphenyl ethers (PBDEs)—both of which are classified as persistent organic pollutants [5]—in addition to heavy metals [6,7], industrial chemicals, and pesticides [8,9]. In communities adjacent to the CSB, especially in El Salto, Jalisco, socio-environmental conflicts have emerged due to the adverse impacts of these pollutants on public health and quality of life [10]. All of these genotoxic contaminants can be associated with an increase in cancer cases.

The existence of genotoxic pollutants in surface waters has been documented in Europe, Asia, and South America [11,12,13,14,15,16]. A clear association between genetic damage and chemical contamination has been reported [13,14,17]. Additionally, access to potable water has been linked to decreased child death rates [18].

Lake Chapala, the largest lake in Mexico, constitutes the primary water source for the Guadalajara metropolitan area. It also functions as a sink for municipal wastewater and industrial effluents. Severe contamination of this lake by heavy metals, pesticides, PCBs, and various other xenobiotic compounds negatively affects native aquatic biota [1,6,9,19,20,21] and presents significant public health risks to the human populations residing near the CSB [10,22]. Many of these communities exhibit high cancer incidence rates; nonetheless, there are no effective governmental interventions in place to mitigate pollution [23,24]. Alvarez-Moya et al. [9] and Rizo-Decelis et al. [25] reported the presence of genotoxic substances in the waters of the Santiago River near El Salto, Jalisco; however, scientific articles addressing the genotoxic potential of these waters are limited and outdated.

The effects of genotoxic agents can be measured using diverse bioassays. For example, the alkaline comet assay is widely utilized as a simple and rapid technique for assessing DNA strand breaks in cells of various organisms, with good sensitivity and repeatability [26,27]. This assay is also widely used in human biomonitoring to both measure DNA damage as a marker of exposure to genotoxic agents and investigate genoprotective effects [28]. Additionally, the comet test detects different types of DNA damage and is relatively easy to apply to any tissue of interest. This approach complements a previous study by Gomez-Meda et al. [6], who used micronuclei to assess heavy metal contamination in the Santiago River. This test is capable of detecting leachate-induced chromosomal instability [29].

Due to severe water pollution, the inadequate efficacy of governmental water treatment strategies, and the associated social issues in the CSB, this study aimed to evaluate the genotoxic potential of water samples taken from Lake Chapala in the communities of Chapala, Ajijic, Jamay, and La Barca and from the Santiago River in El Salto–Juanacatlán (waterfall), Cuenca del Ahogado, and Puente Grande, all situated in the state of Jalisco. Simultaneously, we assessed DNA damage to peripheral blood lymphocytes in individuals residing near these water bodies due to their chronic environmental exposure.

## 2. Materials and Methods

### 2.1. Reagents Used

Ethylmethanesulfonate (EMS) (CAS 66-27-3) were purchased from Sigma Chemical Co. (Guadalajara, Jalisco, México). Both dimethyl sulfoxide (DMSO) (CAS 67-68-5) and disodium salt EDTA (CAS 60-00-4) were obtained from J.T. Baker (Ciudad de México, México).

### 2.2. Collection of Water Samples

Populations: (a) Lake Chapala: Chapala (20°31′10″ N 103°10′41″ W), Ajijic (20°17′58″ N 103°15′29″ W), La Barca (20°17′25″ N 102°32′44″ W), and Jamay (20°17′40″ N 102°42′35″ W); and (b) Santiago River: El Salto–Juanacatlán (waterfall) (20°31′10″ N 103°10′41″ W–20°30′34″ N 103°10′02″ W), Cuenca del Ahogado (20°30′20″ N 103°14′06″ W), and Puente Grande (20°34′11″ N 103°09′14″ W). All populations were located in the state of Jalisco. El Salto and Juanacatlán draw the same water from the Santiago River; however, they are separate sites. Two liters of water were collected in October 2024 in well-cleaned, pre-sterilized glass flasks, which were then sealed and protected with light-protective covers. The samples were transported to the laboratory in under two hours under controlled conditions. To eliminate the possibility of bacteriological contamination, the water was filtered through a 0.22 μm membrane previously sterilized [9,29]. 

### 2.3. Collection of Human Blood Cells

With prior informed consent and in accordance with the guidelines and considerations of the Comisión Nacional de Bioética (National Bioethics Commission), 12 healthy student volunteers under the age of 25 each gave 200 μL of blood via fingertip puncture. The young students who decided to participate in this study were informed about their participation and signed an authorization document (informed consent). Previous exposure to genotoxic agents (exclusion criteria: smoking, under medical treatment, drug consumption, environmental exposure to contaminants) was confirmed using a questionnaire. Blood samples were transferred to Falcon tubes containing 5 mL of physiological saline solution (PSS) and promptly delivered to the laboratory. Tubes were centrifuged at 3000 rpm for 10 min, the supernatant was discarded, and the pellet was homogenized in 1 mL of PSS and stored at 4 °C until further use.

### 2.4. Assessment of the Genotoxic Potential of Water Samples

To avoid cell lysis, 1 mL of 1.8% PSS was mixed with 1 mL of contaminated water. Next, 100 μL of the previously obtained lymphocyte suspension from healthy volunteers was added. The mixtures were incubated at 4 °C for 2 h. The process was conducted separately for each of the 12 healthy participants involved in the study. The same process was carried out using water samples from each of the eight locations while employing the remaining cell suspensions. After the exposure period, mixtures were centrifuged at 3000 rpm for 10 min, and the supernatant was discarded. The pellet was resuspended in 500 μL of standard PSS and stored at 4 °C until the alkaline comet assay.

### 2.5. Assessment of Genetic Damage to Blood Cells in Individuals Exposed to Contaminated Waters

Blood samples from residents living near the river (≤500 m from the water body) were collected from 12 volunteers per population through household visits in Chapala, Ajijic, La Barca, Jamay, El Salto–Juanacatlán, Cuenca del Ahogado, and Puente Grande. who decided to participate in this study were informed about their participation and signed a document (informed consent). Adherent to the protocol prescribed by Alvarez-Moya et al. [9], the study objectives were explained to the participants, and a questionnaire was administered to obtain relevant information regarding medical treatments, health risk factors, age, water contact frequency, self-reported water-related illnesses, and known cancer cases. The exclusion criteria used were those mentioned in the questionnaire: smoking, being under medical treatment, drug use, and living more than 500 m from the Santiago River. Afterward, blood donation was requested, and approximately 100 μL of blood was collected using the same procedure described above, and 1 mL of 0.9% PSS was added to each sample. In this case, no treatment was applied, and the procedure was repeated for each of the 12 participants from each of the eight populations. Both positive (EMS 50 mM) and negative (unexposed blood cells) controls were included. Finally, samples were centrifuged at 3000 rpm for 10 min, the supernatant was removed, and the pellet was resuspended in 500 μL of normal PSS. The suspensions were stored at 4 °C until the alkaline comet assay was performed.

### 2.6. Alkaline Comet Assay

Cell suspensions (human peripheral blood cells from healthy young persons and from exposed residents) were analyzed utilizing the alkaline comet assay, in accordance with the methodology established by Singh et al. [27]. Microscope slides were pre-coated with 1% normal-melting-point (NMP) agarose and permitted to solidify; the layer was later detached to obtain a clean surface. Subsequently, 300 µL of low-melting-point (LMP) agarose 0.7% was applied as the initial layer. A combination of 250 µL of 0.6% LMP agarose and 10 µL of nuclear suspension was added as the second layer, followed by a third layer of 100 µL of 0.6% LMP agarose. Immediately after solidification, slides were washed with distilled water and submerged in a lysis solution (2.5 mM NaCl, 10 mM Na_2_EDTA, 10 mM Tris-HCl, 1% lauroyl sarcosinate, 1% Triton X-100, and 10% DMSO, pH 10) for 24 h at 4 °C. Subsequent to lysis, the slides were positioned in a horizontal electrophoresis chamber filled with alkaline buffer (30 mM NaOH, 1 mM Na_2_EDTA, pH 13) for 45 min at 4 °C. Electrophoresis was conducted for 20 min at 200 mA and 9 V. Slides were subsequently rinsed with distilled water for 1 min and stained with 80 µL of 0.4 µg/mL ethidium bromide for 3 min, followed by two further washes with distilled water. Comets were observed using an Axioskop fluorescent microscope (Gotinga, Germany) equipped with a 560 nm excitation filter. The tail length was measured with the Comet Assay electrophoresis System II software (4250-050-ES) (ZEEIZ SINOPTIC MIKRO S.A DE C.V, Guadalajara, México, 2012). Each experiment was conducted in duplicate; that is, a parallel experiment was carried out.

### 2.7. Statistical Analysis

A minimum of 100 comets per individual was assessed to determine the average DNA migration. Outcomes from exposed groups were compared with both negative and positive controls. Data analysis was performed utilizing Minitab software 22.1. Group variance was evaluated using one-way ANOVA (F-test), because it assumes that all experimental groups have the same behavior, and was followed by Dunnett’s multiple comparison test. A probability level of *p* ≤ 0.05 was considered statistically significant.

## 3. Results

**Figure 1:**
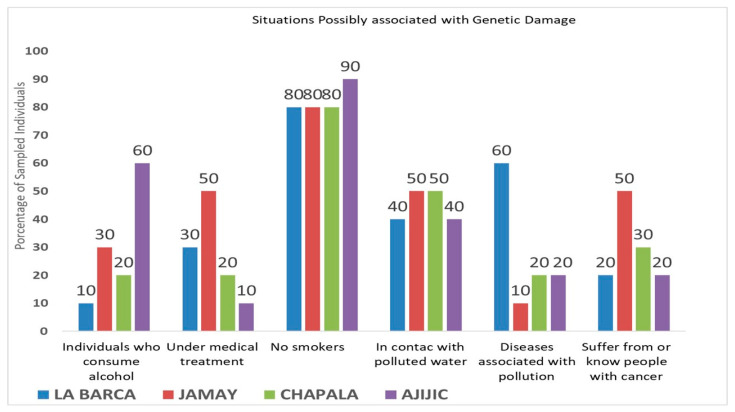
Risk factors linked to the induction of DNA damage and the percentage of individuals (among the surveyed population) found for each risk, according to surveys conducted in La Barca, Jamay, Chapala, and Ajijic. The chart also illustrates the percentage of cancer cases documented or recognized by inhabitants, which they attribute to direct exposure to toxic waters from Lake Chapala.

**Figure 2:**
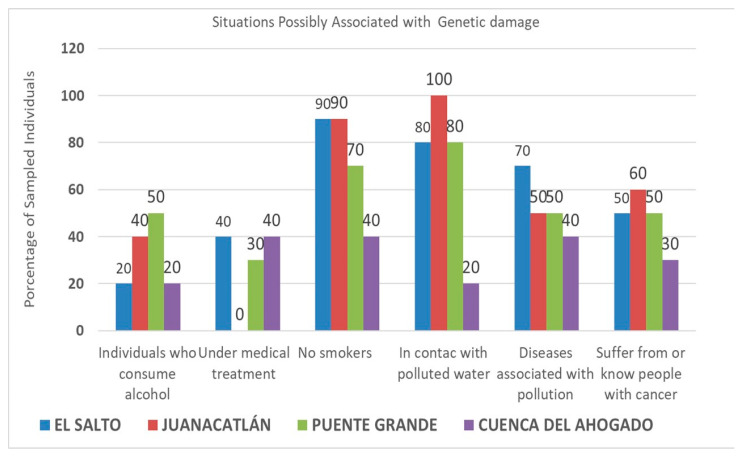
Risk factors linked to the induction of DNA damage and the percentage of individuals (among the surveyed population) identified for each risk, according to surveys conducted in El Salto–Juanacatlán, Puente Grande, and Cuenca del Ahogado. The chart also illustrates the percentage of cancer cases documented or recognized by inhabitants, which they attribute to direct exposure to contaminated waters from the Santiago River.

Survey data from the studied populations regarding risk factors associated with DNA damage, their respective percentages, and reported cancer cases (either personally experienced or known by respondents)—associated with direct contact with the contaminated waters of Lake Chapala and the Santiago River—are illustrated in Figure 1 and Figure 2.

Figure 1 and Figure 2 illustrate significant diversity in the percentage of individuals who use alcohol or are undergoing medical treatment. Individuals who drink alcohol indicated infrequent consumption. The most common medical conditions under treatment encompassed hypertension, diabetes, gastrointestinal disorders, and illnesses requiring antibiotic therapy.

**Figure 3:**
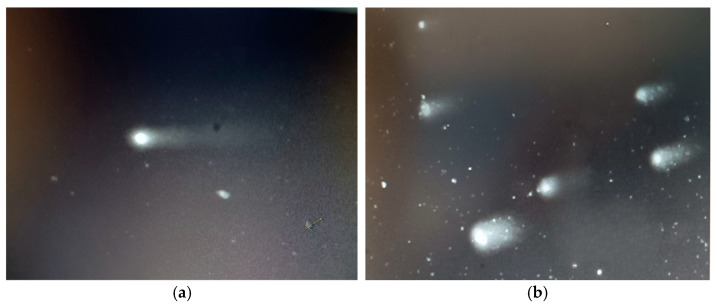
Comets of healthy cells exposed to the contaminated waters of the Santiago River (**a**) and cometized cells of inhabitants living near the waters of the Santiago River (Juanacatlán) (**b**).

**Figure 4:**
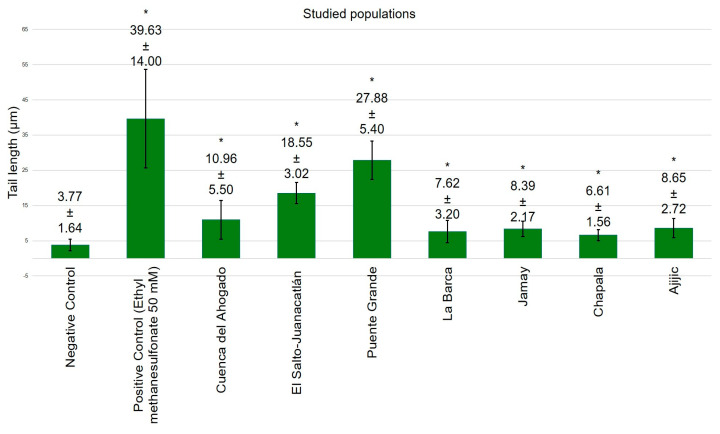
Genotoxic activity of contaminated waters from communities adjacent to the Chapala–Santiago basin in healthy human lymphocytes subjected to in vitro exposure, assessed using the tail length parameter (* *p* ≤ 0.05).

Residents in proximity to Lake Chapala (Figure 1) had low rates of smoking, while the Santiago River region (Figure 2) showed greater variability in smoking rates, with Cuenca del Ahogado reporting the greatest proportion of smokers; still, the daily cigarette consumption of individuals from this area varied significantly. Approximately 40–50% of residents surrounding Lake Chapala reported engaging with its waters (Figure 1), which is in contrast to the 20–100% reported in the Santiago River region. Inhabitants of El Salto and Juanacatlán, Jalisco, reported direct interaction with the water through respiratory or dermal exposure (attributable to soap particles from the Santiago River), with 50–70% associating ailments such as allergies, renal failure, dermatitis, and respiratory diseases with this exposure; meanwhile, 30–60% stated known cancer cases they associate with the contaminated water (Figure 1). On the other hand, 10–60% of inhabitants around Lake Chapala associated illnesses with the lake and claimed a lower incidence of cancer cases (20–50%) (Figure 2). Comets of healthy cells exposed to the contaminated waters of the Santiago River and cometized cells of an individual living near the waters of the Santiago River (Juanacatlán) are presented in Figure 3 and Figure 4.

**Figure 5:**
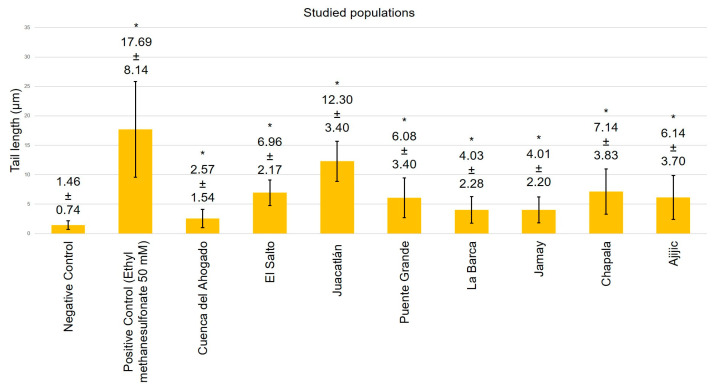
DNA damage observed in lymphocytes from individuals residing within 500 m of the waters of the Chapala–Santiago basin assessed using the tail length parameter (* *p* ≤ 0.05).

**Figure 6:**
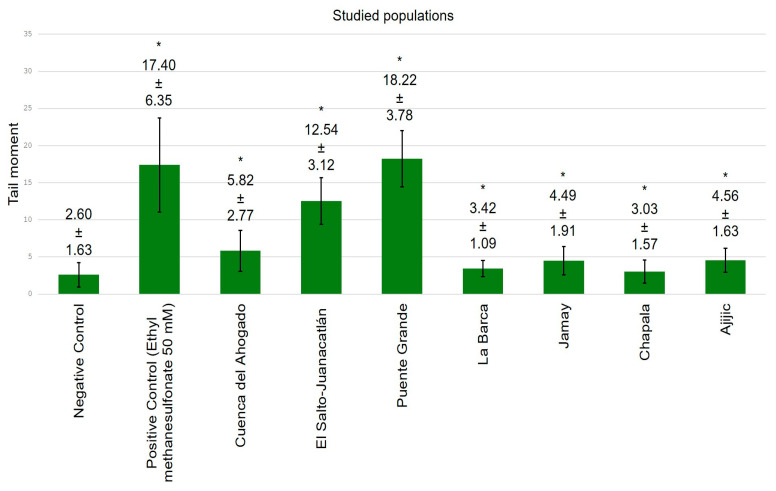
Genotoxic activity of contaminated waters from communities adjacent to the Chapala–Santiago basin in healthy human lymphocytes subjected to in vitro exposure assessed using the tail moment parameter (* *p* ≤ 0.05).

**Figure 7:**
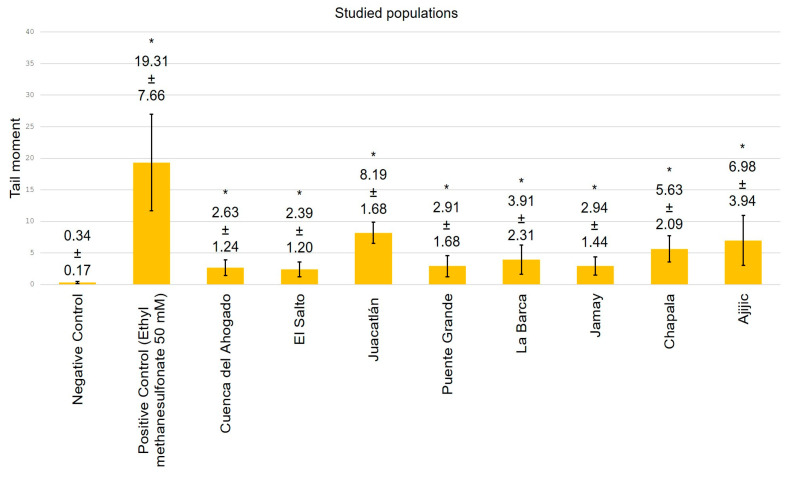
DNA damage observed in lymphocytes from individuals residing within 500 m of the waters of the Chapala–Santiago basin assessed using the tail moment parameter (* *p* ≤ 0.05).

Figure 4, Figure 5, Figure 6 and Figure 7 exhibit data demonstrating the genotoxic activity of contaminated waters in diverse populations within the Chapala–Santiago basin, as well as the DNA damage observed in individuals living near these polluted water bodies. An assessment using the tail length parameter (Figure 4) showed that the average DNA damage (from genotoxic activity of the water) detected in lymphocytes from healthy young donors subjected to in vitro exposure was significantly greater than what was reported in lymphocytes from local residents (Figure 5); although, all values exhibited significant differences (*p* ≤ 0.05) in comparison to their respective negative controls. The elevated genotoxic activity found in lymphocytes subjected in vitro to contaminated waters from Cuenca del Ahogado, El Salto–Juanacatlán (waterfall), and Puente Grande is directly correlated with the most polluted areas. In Cuenca del Ahogado, urban wastewater is produced and subsequently merged with the waters of the Santiago River (in El Salto–Juanacatlán), which are polluted by industrial effluents and pesticides. This pollutant mixing occurs along a 7 km stretch and concludes at Puente Grande, where the waters exhibit their peak genotoxic activity. Meanwhile, the waters from La Barca, Jamay, Chapala, and Ajijic (adjacent to Lake Chapala) exhibited significant differences relative to the negative control (*p* ≤ 0.05); however, their genotoxicity was of lower magnitude compared to what was observed in Santiago River populations (Cuenca del Ahogado, El Salto–Juanacatlán, and Puente Grande).

The DNA damage observed in lymphocytes from inhabitants near contaminated waterways across all zones (Figure 5) exhibited a distinct pattern from the in vitro findings of this study, but it was still significant compared to the negative control (*p* ≤ 0.05). DNA damage was primarily localized in El Salto–Juanacatlán, Puente Grande, and Cuenca del Ahogado. Residents near Lake Chapala exhibited significant DNA damage (*p* ≤ 0.05), comparable to that observed in in vitro studies.

The evaluation utilizing the tail moment parameter demonstrated a similar pattern to that observed with tail length; however, a notable difference was identified in the in vitro analysis (water genotoxicity) for Puente Grande (Figure 4): the extent of DNA damage quantified by tail moment surpassed that of the positive control, a finding not replicated with tail length (Figure 6).

## 4. Discussion

The Lerma–Chapala–Santiago basin is one of the most contaminated regions in México [10] (see Supplementary Material), with its waters exhibiting pollution from heavy metals [30,31], pesticides [7], and both industrial and municipal waste [5,32]. The existence of these pollutants in Lake Chapala is deemed to present a moderate threat to aquatic organisms [33]. Another report suggests that the ingestion of contaminated water results in renal disorders, cancer, and diabetes [34], and inhabitants of communities adjacent to the Chapala–Santiago basin report a significant incidence of illnesses, including cancer, renal and dermatological disorders, allergies, and bronchitis [35]. Therefore, we believe that the risk to human health and biodiversity may exceed the assessments made in prior reports. Moreover, Lake Chapala serves as the principal water source for the Guadalajara metropolitan area [36], while the Santiago River, which originates from this lake, traverses other metropolitan municipalities and functions as a channel for pollutants [10,37]. The findings of the current study suggest that the mutagenic activity of these waters results from agents that can induce genetic instability in human cells and other species, as noted by Alvarez-Moya et al. [9]. This discovery aligns with the increased cancer incidence observed in inhabitants of El Salto–Juanacatlán and Puente Grande, Jalisco [35]. The observed DNA damage arises from the cumulative effects of numerous chemical pollutants accumulated across the basin [23], rather than from a single contaminant.

The water-induced DNA damage observed in populations around Lake Chapala (La Barca, Jamay, Chapala, and Ajijic) is comparable to that reported in the lymphocytes of the local population, although it becomes even more pronounced when assessed using the tail moment parameter. The tail moment provides more consistent estimates of DNA damage due to its reduced variability [38], thus strengthening the evidence that the waters of Chapala and Ajijic present a relatively lower genetic risk compared to those of El Salto–Juanacatlán and Puente Grande. Based on both the tail moment and tail length parameters, the diminished genotoxic activity of waters in Lake Chapala communities relative to the Santiago River can be attributed to the relatively lower presence of genotoxins in Lake Chapala communities, as these locations are situated upstream from the river’s most contaminated segment.

The significant genotoxic activity observed in Puente Grande, El Salto–Juanacatlán, and Cuenca del Ahogado—areas adjacent to the Santiago River—can be ascribed to industrial discharges (in El Salto–Juanacatlán), municipal effluents (in Cuenca del Ahogado), or a combination of both (in Puente Grande) [37], which explains the increased genotoxicity of these waters. These findings align with those of Gómez-Meda et al. [6]. The water collection for this study occurred in October 2024, and when the Santiago River exhibited substantial flow, genotoxicity values persisted at elevated rates; it is probable that the genotoxic risk escalates during the dry season, as previously documented [32]. The establishment of a wastewater treatment plant in 2015 has evidently failed to enhance water quality, with the elevated genotoxicity still presenting a considerable risk to public health and biodiversity.

Analysis of tail length data indicates that DNA damage to human lymphocytes due to direct exposure to water samples escalated progressively in Cuenca del Ahogado, El Salto–Juanacatlán, and Puente Grande (10.96, 18.55, and 27.88 μm, respectively), which exactly correlates with the course of the Santiago River [39]. This suggests that the waters from Cuenca del Ahogado are genotoxic mainly due to municipal effluents from the Guadalajara metropolitan area. The waters converge with the Santiago River near El Salto–Juanacatlán, where they merge with industrial effluents, hence increasing genotoxicity. In Puente Grande, situated 7 km downstream from this point of convergence, the water demonstrates the greatest genotoxic activity. Additional research is required to clarify the connections between various pollutants and the consequent elevations in genotoxic potential. Particularly, in El Salto–Juanacatlán and Puente Grande, tail moment exhibited a similar pattern to tail length; nevertheless, the level of water genotoxicity approached that of the positive control, suggesting the presence of combined genotoxins and the induction of different types of DNA damage.

The slight difference in DNA damage levels between water genotoxicity assays and those seen in people from towns adjacent to Lake Chapala indicates regular and direct contact between people living there and these waters. On the other hand, genotoxicity levels in waters from settlements along the Santiago River (Cuenca del Ahogado, El Salto–Juanacatlán, and Puente Grande) do not align with DNA damage to the lymphocytes of people living there, with the exception of El Salto–Juanacatlán. This trend is associated with the observation that people in Cuenca del Ahogado and Puente Grande avoid direct interaction with the river, but in El Salto–Juanacatlán, substantial amounts of soap particles containing pollutants are disseminated across both communities, forcing direct contact. Residents attribute these particles to various illnesses, including cancer [35,40]. Our findings strongly suggest a correlation between pollutant exposure and DNA damage.

The use of the comet test and the experimental design of this study allowed us to infer the presence of various types of genetic damage, not just chromosomal breaks. Additionally, water samples were evaluated with a wide variety of genotoxicants present, not just heavy metals, as previously reported. Furthermore, it was possible to establish an association between the genotoxic capacity of water bodies and the genetic damage observed in the inhabitants of the studied populations. The application of the surveys allowed us to visualize the relationship between inhabitants’ perception of risk, the quantification of genetic damage in them, and the assessment of the genotoxic capacity of the waters.

Some limitations of this study include inaccurate data reported by residents regarding exposure time (hours per day or years of living in the area), which may lead to some degree of bias in the data. Furthermore, physicochemical measurements were not performed, which may vary from one area to another and could alter genotoxic properties.

Although studies have been reported on the waters of the Santiago River, these were aimed at evaluating the presence and genotoxicity of heavy metals [6] and genetic damage using the comet test [9] before the implementation of a treatment plant 10 years ago. As an update, our data indicates an elevation in genotoxicity of the waters due to the lack of operation of the treatment plant and an increase in chemical pollutants from industrial areas, which can be associated with different types of genetic damage [41,42]. The increase in genotoxics within these waters aligns with the increase in cancer cases in the studied populations [35]. Ruiz-Lara et al. [43], in the Madin Dam (México), reported similar results.

## 5. Conclusions

The waters of the Chapala–Santiago basin demonstrate genotoxic activity that can cause DNA damage in people and other organisms within the basin, presenting a significant threat to biodiversity and the health of people living nearby. The water bodies in this basin serve as effluent disposal sites from industrial facilities and municipal sewage systems. These effluents contain elevated concentrations of genotoxic agents that cause DNA damage linked to an increase in various diseases and an elevated incidence of cancer, especially among the population of El Salto–Juanacatlán. The genetic risk presented by these waters over many seasons demands investigation. It is essential to warn the general public of the hazards linked to exposure to these waters and improve governmental measures to mitigate pollution. Besides technical governmental efforts, actions must be undertaken to deal with the corruption associated with the release of pollutants into these water bodies.

## Data Availability

The data presented in this study are available on request from the corresponding author.

## Supplementary Materials

The following supporting information can be downloaded at https://www.mdpi.com/article/10.3390/toxics13100887/s1. Figure S1: The insulting environmental pollution of the Santiago River, bordering the towns of El Salto, Juanacatlán, and Puente Grande, Jalisco, México. File S1: Informed consent and surveys conducted among residents of municipalities bordering the Santiago River in Jalisco, México.
