# Supplementary material for: Genetic Damage to Human Lymphocytes Induced by Contaminated Water in Populations Surrounding Lake Chapala and the Santiago River, Jalisco, México
Source: Toxics. 2025 Oct 17;13(10):887. doi: 10.3390/toxics13100887 (PMC12568238; doi:10.3390/toxics13100887)
Supplement: Supplementary file 1 [file toxics-13-00887-s001.zip › toxics-3897827-supplementary.pdf]

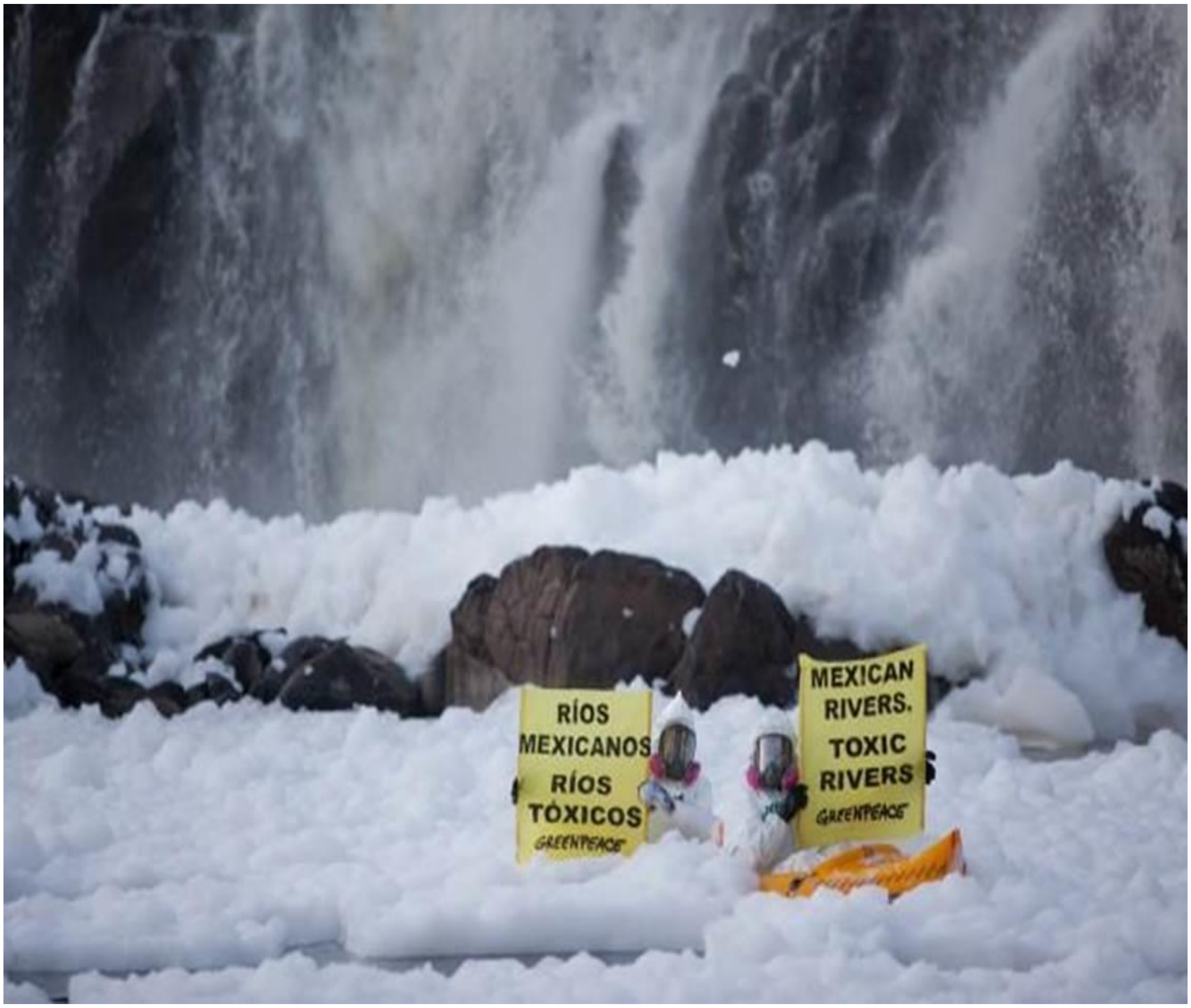

Illustration 1. The insulting environmental pollution of the Santiago River, bordering the towns of El Salto, Juanacatlán and Puente Grande, Jalisco, Mexico.
Fuente: directa [Arellano-Aguilar, Ortega y Gesundheit, 2012]

Encuestas

Ajijic

Consentimiento Informado

Nombre del proyecto en el cual habrá la participación:

Potencial genotóxico de las aguas de el Río Santiago en las poblaciones de El Salto, Juanacatlán, Cuenca del Ahogado, Puente Grande, La Barca y Jamay del estado de Jalisco y daño genético en linfocitos humanos de los habitantes vecinos.

Participantes voluntarios:

Habitantes de zonas aledañas al Río Santiago mayores de 18 años.

Información del proyecto en cual participan:

Práctica: donación de una gota de sangre obtenida por punción anular usando material nuevo y completamente limpio.

Información científica del proyecto a resolver.

Pregunta científica

Las aguas contaminadas del Río Santiago pueden producir mutaciones y cáncer?

Hipótesis:

Los componentes genotóxicos del agua de la cuenca Lerma-Chapala-Santiago poseen actividad genotóxica capaz de inducir daño genético en los habitantes de estas poblaciones detectable mediante la prueba del cometa.

Objetivo

Evaluar el potencial genotóxico de las aguas de el Río Santiago en El Salto del estado de Jalisco, en las poblaciones de La Barca y Jamay y el daño genético en linfocitos humanos de habitantes aledaños.

Decisión de participación

Descripción del proyecto. ¿Qué se va hacer?

Se donará una gota de sangre para evaluar el daño genético en células de sangre de personas expuestas a estas aguas.

Las actividades a realizar por parte de los individuos.

Donación de una gota de sangre periférica mediante punción anular.

Riesgo: No existe.

Manejo de datos. Usados de forma confidencial y para fines científicos.

Aceptación (sí) o **rechazo** (no) para participar.

**Nombre**	**Aceptación** Si	No	**Firma**
Judith Diaz	Si		Diaz Judith DB.
[illegible]	SI		
Juan Manuel Granados	Si		Juan Manuel Granados
Yolanda Castellanos	SI		Yolanda C.
DAVID A. ALVARADO	SI		
Carlos Alberto D [illegible]	YES		
Victor Mendoza	Si		
[illegible]	Si		
Alfredo Salazar	SI		
sheila	Si		

Número telefónico para información relacionada con los resultados: 37771191 ext 33207 Doctor Carlos Álvarez moya

Encuesta Hábitos y salud

Nombre yolanda s castellon

Edad 56

1. **¿Fuma? Sí No** (No underlined)
   Si su respuesta fue sí: ¿Con qué frecuencia de cigarrillos al día?

   ____________ **cigarrillos al día.**

2. **¿Se encuentra tomando algún tratamiento médico? Sí No** (No underlined)
   Si su respuesta fue sí, ¿de qué tipo?

   ____

3. **¿Ingiere bebidas alcohólicas? Sí No** (Sí underlined)
   Si su respuesta fue sí, ¿con qué frecuencia?

   1 cerveza.

   ____________ **días a la semana**

4. **¿Consume o está en contacto con las aguas contaminadas? Sí No** (No underlined)
   ¿en qué forma? ____

5. **¿Tiene algún tipo de padecimiento que pueda asociar al agua contaminada?**
   Sí No (No underlined) **¿Cuál?** ____

6. **¿Usted o alguien cercano a usted tiene cáncer?**
   Sí ______________________ **¿De qué tipo?** ______________________
   No (underlined) ______________________ **¿Qué edad tiene?** ______________________

7. **¿En qué época considera más difícil la contaminación en el río?**

   Cequia.

Encuesta Hábitos y salud

Nombre Juan manuel Granados

Edad 61

1. **¿Fuma? Sí No**

   Si su respuesta fue sí: ¿Con qué frecuencia de cigarrillos al día?

   —

   __________cigarrillos al día.

2. **¿Se encuentra tomando algún tratamiento médico? Sí No**

   Si su respuesta fue sí, ¿de qué tipo?

   —

3. **¿Ingiere bebidas alcohólicas? Sí No**

   Si su respuesta fue sí, ¿con qué frecuencia?

   —días a la semana

4. **¿Consume o está en contacto con las aguas contaminadas? Sí No**

   ¿en qué forma? —

5. **¿Tiene algún tipo de padecimiento que pueda asociar al agua contaminada?**

   Sí No ¿Cuál? —

6. **¿Usted o alguien cercano a usted tiene cáncer?**

   Sí__________ ¿De qué tipo?__________

   No__________ ¿Qué edad tiene?__________

7. **¿En qué época considera más difícil la contaminación en el río?**

   lluvias.

Encuesta Hábitos y salud

Nombre [illegible] alvarado

Edad 73

1. **¿Fuma? Sí No**
   Si su respuesta fue sí: ¿Con qué frecuencia de cigarrillos al día?

   __________cigarrillos al día.

2. **¿Se encuentra tomando algún tratamiento médico? Sí ~~No~~**
   Si su respuesta fue sí, ¿de qué tipo?

   ____

3. **¿Ingiere bebidas alcohólicas? Sí No**
   Si su respuesta fue sí, ¿con qué frecuencia?

   7 **días a la semana**

4. **¿Consume o está en contacto con las aguas contaminadas? Sí No**
   ¿en qué forma?__________________________________

5. **¿Tiene algún tipo de padecimiento que pueda asociar al agua contaminada?**
   Sí No ¿Cuál?__________________________________

6. **¿Usted o alguien cercano a usted tiene cáncer?**
   Sí______________________ **¿De qué tipo?**______________________
   No______________________ **¿Qué edad tiene?**______________________

7. **¿En qué época considera más difícil la contaminación en el río?**

   lluvias.

Encuesta Hábitos y salud

Nombre Jesus sutilio

Edad 51

1. ¿Fuma? Sí No
   Si su respuesta fue sí: ¿Con qué frecuencia de cigarrillos al día?

   __________cigarrillos al día.

2. ¿Se encuentra tomando algún tratamiento médico? Sí No
   Si su respuesta fue sí, ¿de qué tipo?

   ____

3. ¿Ingiere bebidas alcohólicas? Sí No
   Si su respuesta fue sí, ¿con qué frecuencia?

   7 días a la semana

4. ¿Consume o está en contacto con las aguas contaminadas? Sí No
   ¿en qué forma? —

5. ¿Tiene algún tipo de padecimiento que pueda asociar al agua contaminada?
   Sí No ¿Cuál?__________

6. ¿Usted o alguien cercano a usted tiene cáncer?
   Sí__________ ¿De qué tipo?__________
   No__________ ¿Qué edad tiene?__________

7. ¿En qué época considera más difícil la contaminación en el río?
   —

Encuesta Hábitos y salud

Nombre Victor mendoza

Edad 63

1. ¿Fuma? Sí <u>No</u>
   Si su respuesta fue sí: ¿Con qué frecuencia de cigarrillos al día?

   ______ cigarrillos al día.

2. ¿Se encuentra tomando algún tratamiento médico? Sí <u>No</u>
   Si su respuesta fue sí, ¿de qué tipo?

   ____

3. ¿Ingiere bebidas alcohólicas? <u>Sí</u> No
   Si su respuesta fue sí, ¿con qué frecuencia?

   7 días a la semana

4. ¿Consume o está en contacto con las aguas contaminadas? Sí <u>No</u>
   ¿en qué forma? ____________________

5. ¿Tiene algún tipo de padecimiento que pueda asociar al agua contaminada?
   Sí ~~No~~ ¿Cuál? ____________________

6. ¿Usted o alguien cercano a usted tiene cáncer?
   <u>Sí</u> ______ ¿De qué tipo? Madre garganta 75
   Hermano columna 53
   No ______ ¿Qué edad tiene? ______

7. ¿En qué época considera más difícil la contaminación en el río?
   lluvias.

Encuesta Hábitos y salud

Nombre Alfredo Salazar

Edad 38

1. ¿Fuma? Sí <u>No</u>

   Si su respuesta fue sí: ¿Con qué frecuencia de cigarrillos al día?

   __________cigarrillos al día.

2. ¿Se encuentra tomando algún tratamiento médico? Sí <u>No</u>

   Si su respuesta fue sí, ¿de qué tipo?

   ____

3. ¿Ingiere bebidas alcohólicas? Sí <u>No</u>

   Si su respuesta fue sí, ¿con qué frecuencia?

   __________días a la semana

4. ¿Consume o está en contacto con las aguas contaminadas? Sí <u>No</u>

   ¿en qué forma?______________________________

5. ¿Tiene algún tipo de padecimiento que pueda asociar al agua contaminada?

   Sí <u>No</u> ¿Cuál?______________________________

6. ¿Usted o alguien cercano a usted tiene cáncer?

   Sí__________________ ¿De qué tipo?__________________

   <u>No</u>__________________ ¿Qué edad tiene?__________________

7. ¿En qué época considera más difícil la contaminación en el río?

   lluvias.

Encuesta Hábitos y salud

Nombre David Alonso Álvarez

Edad 27

1. **¿Fuma? Sí ~~No~~** (circled: No)
   Si su respuesta fue sí: ¿Con qué frecuencia de cigarrillos al día?

   — **cigarrillos al día.**

2. **¿Se encuentra tomando algún tratamiento médico? Sí No** (circled: No)
   Si su respuesta fue sí, ¿de qué tipo?

   —

3. **¿Ingiere bebidas alcohólicas? Sí No** (circled: No)
   Si su respuesta fue sí, ¿con qué frecuencia?

   — **días a la semana**

4. **¿Consume o está en contacto con las aguas contaminadas? Sí No** (circled: No)
   ¿en qué forma? —

5. **¿Tiene algún tipo de padecimiento que pueda asociar al agua contaminada?**
   Sí No (circled: No) **¿Cuál?**

6. **¿Usted o alguien cercano a usted tiene cáncer?**
   Sí ______ **¿De qué tipo?** —
   No (circled) ______ **¿Qué edad tiene?** —

7. **¿En qué época considera más difícil la contaminación en el río?**
   Tiempo de vacaciones

· Cuidan mucho la zona
· campañas

Encuesta Hábitos y salud

Nombre Sheila

Edad 22

1. **¿Fuma?** **Sí** **(No)** [circled]
   Si su respuesta fue sí: ¿Con qué frecuencia de cigarrillos al día?

   — **cigarrillos al día.**

2. **¿Se encuentra tomando algún tratamiento médico?** **Sí** **(No)** [circled]
   Si su respuesta fue sí, ¿de qué tipo?

   —

3. **¿Ingiere bebidas alcohólicas?** **Sí** **(No)** [circled]
   Si su respuesta fue sí, ¿con qué frecuencia?

   — **días a la semana**

4. **¿Consume o está en contacto con las aguas contaminadas?** **(Sí)** [circled] **No**
   ¿en qué forma? Diariamente

5. **¿Tiene algún tipo de padecimiento que pueda asociar al agua contaminada?**
   Sí **(No)** [circled] **¿Cuál?** —

6. **¿Usted o alguien cercano a usted tiene cáncer?**
   Sí________ **¿De qué tipo?** —
   (No) [circled]________ **¿Qué edad tiene?**________

7. **¿En qué época considera más difícil la contaminación en el río?**
   En tiempo de sequías

Encuesta Hábitos y salud

Nombre Carlos Alberto de la Cruz

Edad 41

1. **¿Fuma?** **(Sí)** **No**

   Si su respuesta fue sí: ¿Con qué frecuencia de cigarrillos al día?

   4 **cigarrillos al día.**

2. **¿Se encuentra tomando algún tratamiento médico?** **Sí** **(No)**

   Si su respuesta fue sí, ¿de qué tipo?

   —

3. **¿Ingiere bebidas alcohólicas?** **(Sí)** **No**

   Si su respuesta fue sí, ¿con qué frecuencia?

   5 **días a la semana**

4. **¿Consume o está en contacto con las aguas contaminadas?** **Sí** **(No)**

   ¿en qué forma? —

5. **¿Tiene algún tipo de padecimiento que pueda asociar al agua contaminada?**

   (Sí) **No** **¿Cuál?** Infecciones diarreas, y de [illegible]

6. **¿Usted o alguien cercano a usted tiene cáncer?**

   Sí______ **¿De qué tipo?** —

   (No)______ **¿Qué edad tiene?** —

7. **¿En qué época considera más difícil la contaminación en el río?**

   Todo el año

   . todo sigue igual

Encuesta Hábitos y salud

Nombre Judith

Edad 22

1. **¿Fuma? Sí No** (No circled)

   Si su respuesta fue sí: ¿Con qué frecuencia de cigarrillos al día?

   — **cigarrillos al día.**

2. **¿Se encuentra tomando algún tratamiento médico? Sí No** (No circled)

   Si su respuesta fue sí, ¿de qué tipo?

   —

3. **¿Ingiere bebidas alcohólicas? Sí No** (Sí circled)

   Si su respuesta fue sí, ¿con qué frecuencia?

   1 **días a la semana**

4. **¿Consume o está en contacto con las aguas contaminadas? Sí No** (Sí circled)

   ¿en qué forma? Directamente

5. **¿Tiene algún tipo de padecimiento que pueda asociar al agua contaminada?**

   Sí No (Sí circled) **¿Cuál?** Alergias

6. **¿Usted o alguien cercano a usted tiene cáncer?**

   Sí ______ **¿De qué tipo?** —

   No (circled) ______ **¿Qué edad tiene?** ______

7. **¿En qué época considera más difícil la contaminación en el río?**

   Todo el año

Encuestas Chapala

Consentimiento Informado

Nombre del proyecto en el cual habrá la participación:

Potencial genotóxico de las aguas de el Río Santiago en las poblaciones de El Salto, Juanacatlán, Cuenca del Ahogado, Puente Grande, La Barca y Jamay del estado de Jalisco y daño genético en linfocitos humanos de los habitantes vecinos.

Participantes voluntarios:

Habitantes de zonas aledañas al Río Santiago mayores de 18 años.

Información del proyecto en cual participan:

Práctica: donación de una gota de sangre obtenida por punción anular usando material nuevo y completamente limpio.

Información científica del proyecto a resolver.

Pregunta científica

Las aguas contaminadas del Río Santiago pueden producir mutaciones y cáncer?

Hipótesis:

Los componentes genotóxicos del agua de la cuenca Lerma-Chapala-Santiago poseen actividad genotóxica capaz de inducir daño genético en los habitantes de estas poblaciones detectable mediante la prueba del cometa.

Objetivo

Evaluar el potencial genotóxico de las aguas de el Río Santiago en El Salto del estado de Jalisco, en las poblaciones de La Barca y Jamay y el daño genético en linfocitos humanos de habitantes aledaños.

Decisión de participación

Descripción del proyecto. ¿Qué se va hacer?

Se donará una gota de sangre para evaluar el daño genético en células de sangre de personas expuestas a estas aguas.

Las actividades a realizar por parte de los individuos.

Donación de una gota de sangre periférica mediante punción anular.

Riesgo: No existe.

Manejo de datos. Usados de forma confidencial y para fines científicos.

Aceptación (sí) o **rechazo** (no) para participar.

**Nombre**	**Aceptación** Si	No	**Firma**
J GADALupe	✓		J GADALUPE
Lourdes Magdalena	✓		Lourdes [illegible]
Margarita Martinez	✓		[illegible]
BIANCA IBARRA	✓		BIANCA IBARRA
Estefania MTZ	✓		Estefania
Ricardo Moreno Jimenez	✓		[illegible]
Yesenia E. Perez	✓		Yesenia E. Perez
Gladis Denisse C.	✓		Gladis Denisse C. C.
Andrés Rojas	✓		Andrés Rojas
Jesus Cervantes C.	✓		Jesus Cervantes C.

Número telefónico para información relacionada con los resultados: 37771191 ext 33207 Doctor Carlos Álvarez moya

Encuesta Hábitos y salud

Nombre Ricardo flores Jimenez

Edad 81

1. **¿Fuma? Sí No**
   Si su respuesta fue sí: ¿Con qué frecuencia de cigarrillos al día?

   — **cigarrillos al día.**

2. **¿Se encuentra tomando algún tratamiento médico? Sí No**
   Si su respuesta fue sí, ¿de qué tipo?

   De los pulmones (alergia)

3. **¿Ingiere bebidas alcohólicas? Sí No**
   Si su respuesta fue sí, ¿con qué frecuencia?

   — **días a la semana**

4. **¿Consume o está en contacto con las aguas contaminadas? Sí No**
   ¿en qué forma? —

5. **¿Tiene algún tipo de padecimiento que pueda asociar al agua contaminada?**
   Sí No ¿Cuál? —

6. **¿Usted o alguien cercano a usted tiene cáncer?**
   Sí________ ¿De qué tipo?________
   No________ ¿Qué edad tiene?________

7. **¿En qué época considera más difícil la contaminación en el río?**
   Todos los años

Encuesta Hábitos y salud

Nombre Andres Rojas

Edad 19

1. ¿Fuma? Sí No
   Si su respuesta fue sí: ¿Con qué frecuencia de cigarrillos al día?

   — cigarrillos al día.

2. ¿Se encuentra tomando algún tratamiento médico? Sí No
   Si su respuesta fue sí, ¿de qué tipo?

   —

3. ¿Ingiere bebidas alcohólicas? Sí No
   Si su respuesta fue sí, ¿con qué frecuencia?

   — días a la semana

4. ¿Consume o está en contacto con las aguas contaminadas? Sí No
   ¿en qué forma? —

5. ¿Tiene algún tipo de padecimiento que pueda asociar al agua contaminada?
   Sí No ¿Cuál? —

6. ¿Usted o alguien cercano a usted tiene cáncer?
   Sí ______ ¿De qué tipo? ______
   No ______ ¿Qué edad tiene? ______

7. ¿En qué época considera más difícil la contaminación en el río?
   Actualmente.

Encuesta Hábitos y salud

Nombre viscenia perez

Edad 36

1. ¿Fuma? Sí No
   Si su respuesta fue sí: ¿Con qué frecuencia de cigarrillos al día?

   — cigarrillos al día.

2. ¿Se encuentra tomando algún tratamiento médico? Sí No
   Si su respuesta fue sí, ¿de qué tipo?

   —

3. ¿Ingiere bebidas alcohólicas? Sí No
   Si su respuesta fue sí, ¿con qué frecuencia?

   — días a la semana

4. ¿Consume o está en contacto con las aguas contaminadas? Sí No
   ¿en qué forma? —

5. ¿Tiene algún tipo de padecimiento que pueda asociar al agua contaminada?
   Sí No ¿Cuál? —

6. ¿Usted o alguien cercano a usted tiene cáncer?
   Sí______ ¿De qué tipo?______
   No______ ¿Qué edad tiene?______

7. ¿En qué época considera más difícil la contaminación en el río?
   En esta epoca.

Encuesta Hábitos y salud

Nombre Gladis Denisse

Edad 27

1. ¿Fuma? Sí No
   Si su respuesta fue sí: ¿Con qué frecuencia de cigarrillos al día?

   — cigarrillos al día.

2. ¿Se encuentra tomando algún tratamiento médico? Sí No
   Si su respuesta fue sí, ¿de qué tipo?

   —

3. ¿Ingiere bebidas alcohólicas? Sí No
   Si su respuesta fue sí, ¿con qué frecuencia?

   1 al mes días a la semana

4. ¿Consume o está en contacto con las aguas contaminadas? Sí No
   ¿en qué forma? —

5. ¿Tiene algún tipo de padecimiento que pueda asociar al agua contaminada?
   Sí No ¿Cuál? —

6. ¿Usted o alguien cercano a usted tiene cáncer?
   Sí ______ ¿De qué tipo? ______
   No ______ ¿Qué edad tiene? ______

7. ¿En qué época considera más difícil la contaminación en el río?
   El año pasado

Encuesta Hábitos y salud

Nombre Jesus servantes

Edad 55

1. **¿Fuma? Sí No**
   Si su respuesta fue sí: ¿Con qué frecuencia de cigarrillos al día?

   — **cigarrillos al día.**

2. **¿Se encuentra tomando algún tratamiento médico? Sí No**
   Si su respuesta fue sí, ¿de qué tipo?

   —

3. **¿Ingiere bebidas alcohólicas? Sí No**
   Si su respuesta fue sí, ¿con qué frecuencia?

   1 **días a la semana**

4. **¿Consume o está en contacto con las aguas contaminadas? Sí No**
   ¿en qué forma? En el pez.

5. **¿Tiene algún tipo de padecimiento que pueda asociar al agua contaminada?**
   Sí No ¿Cuál? —

6. **¿Usted o alguien cercano a usted tiene cáncer?** Mamá hígado 75
   Sí ______ **¿De qué tipo?** hermana colon ~~48~~ 54
   No ______ **¿Qué edad tiene?** ______

7. **¿En qué época considera más difícil la contaminación en el río?**

   Todos.

Encuesta Hábitos y salud

Nombre Blanca [illegible]

Edad 55

1. ¿Fuma? Sí No (circled: No)
   Si su respuesta fue sí: ¿Con qué frecuencia de cigarrillos al día?

   — cigarrillos al día.

2. ¿Se encuentra tomando algún tratamiento médico? Sí No (circled: Sí)
   Si su respuesta fue sí, ¿de qué tipo?

   Hipertensión

3. ¿Ingiere bebidas alcohólicas? Sí No (circled: No)
   Si su respuesta fue sí, ¿con qué frecuencia?

   — días a la semana

4. ¿Consume o está en contacto con las aguas contaminadas? Sí No (circled: Sí)
   ¿en qué forma? Directo

5. ¿Tiene algún tipo de padecimiento que pueda asociar al agua contaminada?
   Sí No ¿Cuál? Insuficiencia Renal (circled: Sí)

6. ¿Usted o alguien cercano a usted tiene cáncer?
   Sí ¿De qué tipo? [illegible] (circled: Sí)
   No ¿Qué edad tiene? 75

7. ¿En qué época considera más difícil la contaminación en el río?
   Tiempos de secas

Encuesta Hábitos y salud

Nombre Estefania

Edad 25

1. ¿Fuma? Sí (No)

   Si su respuesta fue sí: ¿Con qué frecuencia de cigarrillos al día?

   — cigarrillos al día.

2. ¿Se encuentra tomando algún tratamiento médico? Sí (No)

   Si su respuesta fue sí, ¿de qué tipo?

   —

3. ¿Ingiere bebidas alcohólicas? Sí (No)

   Si su respuesta fue sí, ¿con qué frecuencia?

   — días a la semana

4. ¿Consume o está en contacto con las aguas contaminadas? (Sí) No

   ¿en qué forma? Diariamente

5. ¿Tiene algún tipo de padecimiento que pueda asociar al agua contaminada?

   Sí (No) ¿Cuál? —

6. ¿Usted o alguien cercano a usted tiene cáncer?

   Sí___ ¿De qué tipo? —

   (No)___ ¿Qué edad tiene? —

7. ¿En qué época considera más difícil la contaminación en el río?

   Epoca de lluvia

Encuesta Hábitos y salud

Nombre Emilio Cruz

Edad 58

1. ¿Fuma? (Sí) No

   Si su respuesta fue sí: ¿Con qué frecuencia de cigarrillos al día?

   5 cigarrillos al día.

2. ¿Se encuentra tomando algún tratamiento médico? (Sí) No

   Si su respuesta fue sí, ¿de qué tipo?

   Musculo

3. ¿Ingiere bebidas alcohólicas? Sí (No)

   Si su respuesta fue sí, ¿con qué frecuencia?

   — días a la semana

4. ¿Consume o está en contacto con las aguas contaminadas? (Sí) No

   ¿en qué forma? Directa

5. ¿Tiene algún tipo de padecimiento que pueda asociar al agua contaminada?

   Sí (No) ¿Cuál? —

6. ¿Usted o alguien cercano a usted tiene cáncer?

   (Sí)______ ¿De qué tipo? Garganta

   No______ ¿Qué edad tiene? 70 años

7. ¿En qué época considera más difícil la contaminación en el río?

   No lo considera

Encuesta Hábitos y salud

Nombre Lourdes Magdalena Flores

Edad 27

1. **¿Fuma?** **Sí** (circled) **No**

   Si su respuesta fue sí: ¿Con qué frecuencia de cigarrillos al día?

   4 **cigarrillos al día.**

2. **¿Se encuentra tomando algún tratamiento médico?** **Sí** **No** (circled)

   Si su respuesta fue sí, ¿de qué tipo?

   —

3. **¿Ingiere bebidas alcohólicas?** **Sí** **No** (circled)

   Si su respuesta fue sí, ¿con qué frecuencia?

   — **días a la semana**

4. **¿Consume o está en contacto con las aguas contaminadas?** **Sí** (circled) **No**

   ¿en qué forma? Directamente

5. **¿Tiene algún tipo de padecimiento que pueda asociar al agua contaminada?**

   Sí **No** (circled) **¿Cuál?** —

6. **¿Usted o alguien cercano a usted tiene cáncer?**

   Sí______ **¿De qué tipo?** —

   No (circled)______ **¿Qué edad tiene?** —

7. **¿En qué época considera más difícil la contaminación en el río?**

   No lo considera

Encuesta Hábitos y salud

Nombre Margarita Martinez

Edad 50

1. **¿Fuma? Sí (No)**

   Si su respuesta fue sí: ¿Con qué frecuencia de cigarrillos al día?

   — **cigarrillos al día.**

2. **¿Se encuentra tomando algún tratamiento médico? Sí (No)**

   Si su respuesta fue sí, ¿de qué tipo?

   —

3. **¿Ingiere bebidas alcohólicas? Sí (No)**

   Si su respuesta fue sí, ¿con qué frecuencia?

   — **días a la semana**

4. **¿Consume o está en contacto con las aguas contaminadas? (Sí) No**

   ¿en qué forma? Directamente

5. **¿Tiene algún tipo de padecimiento que pueda asociar al agua contaminada?**

   (Sí) No ¿Cuál? Dengue, Alergias

6. **¿Usted o alguien cercano a usted tiene cáncer?**

   Sí ______ **¿De qué tipo?** —

   (No) ______ **¿Qué edad tiene?** ______

7. **¿En qué época considera más difícil la contaminación en el río?**

   Todo el año

Encuestas

Cuenca del Ahogado

Consentimiento Informado

Nombre del proyecto en el cual habrá la participación:

Potencial genotóxico de las aguas de el Río Santiago en las poblaciones de El Salto, Juanacatlán, Cuenca del Ahogado, Puente Grande, La Barca y Jamay del estado de Jalisco y daño genético en linfocitos humanos de los habitantes vecinos.

Participantes voluntarios:

Habitantes de zonas aledañas al Río Santiago mayores de 18 años.

Información del proyecto en cual participan:

Práctica: donación de una gota de sangre obtenida por punción anular usando material nuevo y completamente limpio.

Información científica del proyecto a resolver.

<u>Pregunta científica</u>

Las aguas contaminadas del Río Santiago pueden producir mutaciones y cáncer?

<u>Hipótesis:</u>

Los componentes genotóxicos del agua de la cuenca Lerma-Chapala-Santiago poseen actividad genotóxica capaz de inducir daño genético en los habitantes de estas poblaciones detectable mediante la prueba del cometa.

<u>Objetivo</u>

Evaluar el potencial genotóxico de las aguas de el Río Santiago en El Salto del estado de Jalisco, en las poblaciones de La Barca y Jamay y el daño genético en linfocitos humanos de habitantes aledaños.

Decisión de participación

Descripción del proyecto. ¿Qué se va hacer?

Se donará una gota de sangre para evaluar el daño genético en células de sangre de personas expuestas a estas aguas.

Las actividades a realizar por parte de los individuos.

Donación de una gota de sangre periférica mediante punción anular.

Riesgo: No existe.

Manejo de datos. Usados de forma confidencial y para fines científicos.

Aceptación (sí) o **rechazo** (no) para participar.

Nombre	Aceptación Si	Aceptación No	Firma
Evangelina López	✓		[illegible]
Jesus Emmanuel Mendoza	/		Jesus
Santiago [illegible]	/		[illegible]
Eliceo Jiménez Nuñez	/		[illegible]
J Jesus Gonzalez Loza	/		J Jesus Gonzalez Loza
Maria Rodriguez	/		maria Rodriguez
Francisco Rojas Sotelo	/		Francisco Rojas Sotelo
Santiago Cervantes U	/		[illegible]
M Isabel	✓		[illegible]
J. Emmanuel M.	✓		J. Emmanuel M.

Número telefónico para información relacionada con los resultados: 37771191 ext 33207 Doctor Carlos Álvarez moya

Encuesta Hábitos y salud

Nombre María Isabel Ríos

Edad 68

1. ¿Fuma? (Sí) No
   Si su respuesta fue sí: ¿Con qué frecuencia de cigarrillos al día?

   1 cigarrillos al día.

2. ¿Se encuentra tomando algún tratamiento médico? Sí (No)
   Si su respuesta fue sí, ¿de qué tipo?

   —

3. ¿Ingiere bebidas alcohólicas? (Sí) No
   Si su respuesta fue sí, ¿con qué frecuencia?

   1 días a la semana

4. ¿Consume o está en contacto con las aguas contaminadas? (Sí) No
   ¿en qué forma? Directamente (paso muy seguido)

5. ¿Tiene algún tipo de padecimiento que pueda asociar al agua contaminada?
   Sí No ¿Cuál? ______

6. ¿Usted o alguien cercano a usted tiene cáncer?
   (Sí) ______ ¿De qué tipo? Sangre
   No ______ ¿Qué edad tiene? 63

7. ¿En qué época considera más difícil la contaminación en el río?
   Todo el año

Encuesta Hábitos y salud

Nombre Evangelina López Carbajal

Edad 64

1. **¿Fuma?** **Sí** (circled) **No**

   Si su respuesta fue sí: ¿Con qué frecuencia de cigarrillos al día?

   2 **cigarrillos al día.**

2. **¿Se encuentra tomando algún tratamiento médico?** **Sí** **No** (circled)

   Si su respuesta fue sí, ¿de qué tipo?

   —

3. **¿Ingiere bebidas alcohólicas?** **Sí** (circled) **No**

   Si su respuesta fue sí, ¿con qué frecuencia?

   1 **días a la semana**

4. **¿Consume o está en contacto con las aguas contaminadas?** **Sí** **No** (circled)

   ¿en qué forma? —

5. **¿Tiene algún tipo de padecimiento que pueda asociar al agua contaminada?**

   Sí (circled) **No** **¿Cuál?** Dermatológicos, respiratorios, estomacales

6. **¿Usted o alguien cercano a usted tiene cáncer?**

   Sí ______ **¿De qué tipo?** —

   No (circled) ______ **¿Qué edad tiene?** —

7. **¿En qué época considera más difícil la contaminación en el río?**

   todo el año

- Propuestas sin cumplimiento
- Ninguna casa

Encuesta Hábitos y salud

Nombre Jesús Emanuel Mendoza

Edad 26

1. **¿Fuma? Sí No** (No circled)

   Si su respuesta fue sí: ¿Con qué frecuencia de cigarrillos al día?

   — **cigarrillos al día.**

2. **¿Se encuentra tomando algún tratamiento médico? Sí No** (No circled)

   Si su respuesta fue sí, ¿de qué tipo?

   —

3. **¿Ingiere bebidas alcohólicas? Sí No** (No circled)

   Si su respuesta fue sí, ¿con qué frecuencia?

   — **días a la semana**

4. **¿Consume o está en contacto con las aguas contaminadas? Sí No** (No circled)

   ¿en qué forma? —

5. **¿Tiene algún tipo de padecimiento que pueda asociar al agua contaminada?**

   Sí No (Sí circled) **¿Cuál?** Dengue

6. **¿Usted o alguien cercano a usted tiene cáncer?**

   Sí (circled) **¿De qué tipo?** Estomago

   No **¿Qué edad tiene?** 22

7. **¿En qué época considera más difícil la contaminación en el río?**

   todo el año

- No han hecho nada
- Intentaron para un basurero (2024)
-

Encuesta Hábitos y salud

Nombre Angel Rodriguez

Edad 64

1. **¿Fuma? Sí ~~No~~** (No circled)
   Si su respuesta fue sí: ¿Con qué frecuencia de cigarrillos al día?

   — **cigarrillos al día.**

2. **¿Se encuentra tomando algún tratamiento médico? Sí No** (No circled)
   Si su respuesta fue sí, ¿de qué tipo?

   —

3. **¿Ingiere bebidas alcohólicas? Sí No** (No circled)
   Si su respuesta fue sí, ¿con qué frecuencia?

   — **días a la semana**

4. **¿Consume o está en contacto con las aguas contaminadas? Sí No** (Sí circled)
   ¿en qué forma? Directo

5. **¿Tiene algún tipo de padecimiento que pueda asociar al agua contaminada?**
   Sí No (Sí circled) **¿Cuál?** Alergias, cutáneas, pulmonares

6. **¿Usted o alguien cercano a usted tiene cáncer?**
   Sí____ **¿De qué tipo?** —
   No (circled) ____ **¿Qué edad tiene?** —

7. **¿En qué época considera más difícil la contaminación en el río?**

Todo el año

Encuesta Hábitos y salud

Nombre Santiago Servantes

Edad 57

1. **¿Fuma? Sí No**

   Si su respuesta fue sí: ¿Con qué frecuencia de cigarrillos al día?

   3-5-10 **cigarrillos al día.**

2. **¿Se encuentra tomando algún tratamiento médico? Sí No**

   Si su respuesta fue sí, ¿de qué tipo?

   —

3. **¿Ingiere bebidas alcohólicas? Sí No**

   Si su respuesta fue sí, ¿con qué frecuencia?

   — **días a la semana**

4. **¿Consume o está en contacto con las aguas contaminadas? Sí No**

   ¿en qué forma? —

5. **¿Tiene algún tipo de padecimiento que pueda asociar al agua contaminada?**

   Sí No ¿Cuál? — alergicas diarreicas

6. **¿Usted o alguien cercano a usted tiene cáncer?**

   Sí ______ **¿De qué tipo?** ______

   No ______ **¿Qué edad tiene?** ______

7. **¿En qué época considera más difícil la contaminación en el río?**

   Secas.

Encuesta Hábitos y salud

Nombre Francisco Rojas Sotelo

Edad 62

1. **¿Fuma? Sí No**
   Si su respuesta fue sí: ¿Con qué frecuencia de cigarrillos al día?

   ______ **cigarrillos al día.**

2. **¿Se encuentra tomando algún tratamiento médico? Sí No**
   Si su respuesta fue sí, ¿de qué tipo?

   Dolor paracetamol.

3. **¿Ingiere bebidas alcohólicas? Sí No**
   Si su respuesta fue sí, ¿con qué frecuencia?

   ______ **días a la semana**

4. **¿Consume o está en contacto con las aguas contaminadas? Sí No**
   ¿en qué forma? ______

5. **¿Tiene algún tipo de padecimiento que pueda asociar al agua contaminada?**
   Sí No ¿Cuál? ______

6. **¿Usted o alguien cercano a usted tiene cáncer?**
   Sí ______ **¿De qué tipo?** Primo
   No ______ **¿Qué edad tiene?** 64.

7. **¿En qué época considera más difícil la contaminación en el río?**
   Todo el año.

Encuesta Hábitos y salud

Nombre Miguel angel naranjo

Edad 66

1. **¿Fuma? Sí No**
   Si su respuesta fue sí: ¿Con qué frecuencia de cigarrillos al día?

   __________cigarrillos al día.

2. **¿Se encuentra tomando algún tratamiento médico? Sí No**
   Si su respuesta fue sí, ¿de qué tipo?

   Cabeza.

3. **¿Ingiere bebidas alcohólicas? Sí No**
   Si su respuesta fue sí, ¿con qué frecuencia?

   __________días a la semana

4. **¿Consume o está en contacto con las aguas contaminadas? Sí No**
   ¿en qué forma? —

5. **¿Tiene algún tipo de padecimiento que pueda asociar al agua contaminada?**
   Sí No ¿Cuál?__________

6. **¿Usted o alguien cercano a usted tiene cáncer?**
   Sí__________ **¿De qué tipo?**__________
   No__________ **¿Qué edad tiene?**__________

7. **¿En qué época considera más difícil la contaminación en el río?**
   Secas.

Encuesta Hábitos y salud

Nombre Eliseo Jimenez

Edad 73

1. ¿Fuma? Sí No
   Si su respuesta fue sí: ¿Con qué frecuencia de cigarrillos al día?

   casi todo el dia cigarrillos al día.

2. ¿Se encuentra tomando algún tratamiento médico? Sí No
   Si su respuesta fue sí, ¿de qué tipo?

   Dolor de rodilla

3. ¿Ingiere bebidas alcohólicas? Sí No
   Si su respuesta fue sí, ¿con qué frecuencia?

   __________días a la semana

4. ¿Consume o está en contacto con las aguas contaminadas? Sí No
   ¿en qué forma? —

5. ¿Tiene algún tipo de padecimiento que pueda asociar al agua contaminada?
   Sí No ¿Cuál?__________

6. ¿Usted o alguien cercano a usted tiene cáncer?
   Sí__________ ¿De qué tipo?__________
   No__________ ¿Qué edad tiene?__________

7. ¿En qué época considera más difícil la contaminación en el río?
   Todo el año.

Encuesta Hábitos y salud

Nombre J. Jesus Gonzalez

Edad 74

1. **¿Fuma? Sí No**

   Si su respuesta fue sí: ¿Con qué frecuencia de cigarrillos al día?

   ______ **cigarrillos al día.**

2. **¿Se encuentra tomando algún tratamiento médico? Sí No**

   Si su respuesta fue sí, ¿de qué tipo?

   Edad

3. **¿Ingiere bebidas alcohólicas? Sí No**

   Si su respuesta fue sí, ¿con qué frecuencia?

   — **días a la semana**

4. **¿Consume o está en contacto con las aguas contaminadas? Sí No**

   ¿en qué forma? ______

5. **¿Tiene algún tipo de padecimiento que pueda asociar al agua contaminada?**

   Sí No ¿Cuál? ______

6. **¿Usted o alguien cercano a usted tiene cáncer?**

   Sí ______ **¿De qué tipo?** ______

   No ______ **¿Qué edad tiene?** ______

7. **¿En qué época considera más difícil la contaminación en el río?**

   lluvias

Encuesta Hábitos y salud

Nombre María.

Edad 57

1. ¿Fuma? Sí No
   Si su respuesta fue sí: ¿Con qué frecuencia de cigarrillos al día?

   — cigarrillos al día.

2. ¿Se encuentra tomando algún tratamiento médico? Sí No
   Si su respuesta fue sí, ¿de qué tipo?

   —

3. ¿Ingiere bebidas alcohólicas? Sí No
   Si su respuesta fue sí, ¿con qué frecuencia?

   ________ días a la semana

4. ¿Consume o está en contacto con las aguas contaminadas? Sí No
   ¿en qué forma?________

5. ¿Tiene algún tipo de padecimiento que pueda asociar al agua contaminada?
   Sí No ¿Cuál?________

6. ¿Usted o alguien cercano a usted tiene cáncer?
   Sí________ ¿De qué tipo?________
   No________ ¿Qué edad tiene?________

7. ¿En qué época considera más difícil la contaminación en el río?
   Todo el año

Encuestas

Jamay

✓

Consentimiento Informado

Nombre del proyecto en el cual habrá la participación:

Potencial genotóxico de las aguas de el Río Santiago en las poblaciones de El Salto, Juanacatlán, Cuenca del Ahogado, Puente Grande, La Barca y Jamay del estado de Jalisco y daño genético en linfocitos humanos de los habitantes vecinos.

Participantes voluntarios:

Habitantes de zonas aledañas al Río Santiago mayores de 18 años.

Información del proyecto en cual participan:

Práctica: donación de una gota de sangre obtenida por punción anular usando material nuevo y completamente limpio.

Información científica del proyecto a resolver.

<u>Pregunta científica</u>

Las aguas contaminadas del Río Santiago pueden producir mutaciones y cáncer?

<u>Hipótesis:</u>

Los componentes genotóxicos del agua de la cuenca Lerma-Chapala-Santiago poseen actividad genotóxica capaz de inducir daño genético en los habitantes de estas poblaciones detectable mediante la prueba del cometa.

<u>Objetivo</u>

Evaluar el potencial genotóxico de las aguas de el Río Santiago en El Salto del estado de Jalisco, en las poblaciones de La Barca y Jamay y el daño genético en linfocitos humanos de habitantes aledaños.

Decisión de participación

Descripción del proyecto. ¿Qué se va hacer?

Se donará una gota de sangre para evaluar el daño genético en células de sangre de personas expuestas a estas aguas.

Las actividades a realizar por parte de los individuos.

Donación de una gota de sangre periférica mediante punción anular.

Riesgo: No existe.

Manejo de datos. Usados de forma confidencial y para fines científicos.

Aceptación (sí) o **rechazo** (no) para participar.

Nombre	Aceptación Si	Aceptación No	Firma
Alexa Gonzales	✓		Alexa Glz.
Patricia Gonzalez Z.	✓		Patricia G.Z.
Isabel Flores	✓		Isabel. FP
Jennifer Jimenez	✓		[illegible]
Ramiro Sanchez	✓		[illegible]
Ruben Ramos Ulloa	✓		[illegible]
Adrian Hernandez	✓		[illegible]
David Partida	✓		[illegible]
Aurelio Rodriguez A	✓	[illegible]	[illegible]
J Jesús Romero	✓		[illegible]

Número telefónico para información relacionada con los resultados: 37771191 ext 33207 Doctor Carlos Álvarez moya

Encuesta Hábitos y salud

Nombre 1° Jesus Romero Jimenez

Edad 82

1. ¿Fuma? Sí (No)

   Si su respuesta fue sí: ¿Con qué frecuencia de cigarrillos al día?

   ___ cigarrillos al día.

2. ¿Se encuentra tomando algún tratamiento médico? (Sí) No

   Si su respuesta fue sí, ¿de qué tipo?

   hipertension - hidralazina
   - homeprazol

3. ¿Ingiere bebidas alcohólicas? Sí (No)

   Si su respuesta fue sí, ¿con qué frecuencia?

   ___ días a la semana

4. ¿Consume o está en contacto con las aguas contaminadas? (Sí) No

   ¿en qué forma? Va caminar. 10 años
   Cultivos.

5. ¿Tiene algún tipo de padecimiento que pueda asociar al agua contaminada?

   (Sí) No ¿Cuál? garganta / irrita.

6. ¿Usted o alguien cercano a usted tiene cáncer?

   (Sí) ___ ¿De qué tipo? pecho... 20 años.

   No ___ ¿Qué edad tiene? ___

7. ¿En qué época considera más difícil la contaminación en el río?

   Tiempo calor

Encuesta Hábitos y salud

Nombre Rúben Ramos Velasco

Edad 72

1. **¿Fuma? Sí No** (No circled)

   Si su respuesta fue sí: ¿Con qué frecuencia de cigarrillos al día?

   ______ **cigarrillos al día.**

2. **¿Se encuentra tomando algún tratamiento médico? Sí No** (Sí circled)

   Si su respuesta fue sí, ¿de qué tipo?

   Azipril, dosliquz, dolaneurobion

3. **¿Ingiere bebidas alcohólicas? Sí No** (Sí circled)

   Si su respuesta fue sí, ¿con qué frecuencia?

   1 **días a la semana**

4. **¿Consume o está en contacto con las aguas contaminadas? Sí No**

   ¿en qué forma? Caminata por el lago

5. **¿Tiene algún tipo de padecimiento que pueda asociar al agua contaminada?**

   Sí No ¿Cuál? Colesterol, presión arterial

6. **¿Usted o alguien cercano a usted tiene cáncer?**

   Sí ______ **¿De qué tipo?** ______

   No ninguno **¿Qué edad tiene?** ______

7. **¿En qué época considera más difícil la contaminación en el río?**

   Contaminado, 15 años antes estaba limpio. Mal olor. Sigue en pesca, agarran muy adentro, cuando abren compuertas el agua que viene esta bien, lo que huele mal es lo que esta en el canal

Encuesta Hábitos y salud

Nombre Ramiro Sanchez Contreras

Edad 61

1. **¿Fuma? Sí No** (circled: No)

   Si su respuesta fue sí: ¿Con qué frecuencia de cigarrillos al día?

   ______ **cigarrillos al día.**

2. **¿Se encuentra tomando algún tratamiento médico? Sí No** (circled: Sí)

   Si su respuesta fue sí, ¿de qué tipo?

   hipertensión

3. **¿Ingiere bebidas alcohólicas? Sí No** (circled: No)

   Si su respuesta fue sí, ¿con qué frecuencia?

   ______ **días a la semana**

4. **¿Consume o está en contacto con las aguas contaminadas? Sí No** (circled: Sí)

   ¿en qué forma? Pesca y camina (fue pescador) x 8 desde los 8 años

5. **¿Tiene algún tipo de padecimiento que pueda asociar al agua contaminada?**

   Sí No (circled: No) **¿Cuál?** ______

6. **¿Usted o alguien cercano a usted tiene cáncer?**

   Sí (circled) Su papás. **¿De qué tipo?** pancreas y pecho.

   No ______ **¿Qué edad tiene?** ______

7. **¿En qué época considera más difícil la contaminación en el río?**

   Todo el año.

   392 22 10 253.

• Ya casi no hay peces.

• Cuando llueve tiran

Encuesta Hábitos y salud

Nombre Aurelio Gódinez Ortega

Edad 90

1. **¿Fuma? Sí (No)**
   Si su respuesta fue sí: ¿Con qué frecuencia de cigarrillos al día?

   ______ **cigarrillos al día.**

2. **¿Se encuentra tomando algún tratamiento médico? (Sí) No**
   Si su respuesta fue sí, ¿de qué tipo?

   Homeopatia

3. **¿Ingiere bebidas alcohólicas? Sí (No)**
   Si su respuesta fue sí, ¿con qué frecuencia?

   ______ **días a la semana**

4. **¿Consume o está en contacto con las aguas contaminadas? Sí No**
   ¿en qué forma? Paso en bicicleta

5. **¿Tiene algún tipo de padecimiento que pueda asociar al agua contaminada?**
   Sí No ¿Cuál? artritis

6. **¿Usted o alguien cercano a usted tiene cáncer?**
   Sí Vecina con cancer **¿De qué tipo?** ______
   No - **¿Qué edad tiene?** ______

★1958 ~~bajo mucho~~ subio mucho el nivel

★Aparecieron animales muertos despues de contaminación

7. **¿En qué época considera más difícil la contaminación en el río?**
   Muy sucio, antes el nivel del agua llegaba hasta la plaza, olor fetido el de ocotlan y los demas los vierten drenajes. habia pescado blanco antes, popocha tambien, huachinango bagre, hace 50 años metieron bagre y se acabo lo demás. Venden pescado aún. El Lerma y Santiago vienen sucios. Sembradios antes. Se secaron rios aledaños primeros. Se cultivaba frijol y trigo antes

Encuesta Hábitos y salud

Nombre DAVID PARTIDA

Edad 37

1. **¿Fuma? Sí ~~No~~**

   Si su respuesta fue sí: ¿Con qué frecuencia de cigarrillos al día?

   __________ **cigarrillos al día.**

2. **¿Se encuentra tomando algún tratamiento médico? ~~Sí~~ No**

   Si su respuesta fue sí, ¿de qué tipo?

   HOMEOPATICO

3. **¿Ingiere bebidas alcohólicas? Sí ~~No~~**

   Si su respuesta fue sí, ¿con qué frecuencia?

   __________ **días a la semana**

4. **¿Consume o está en contacto con las aguas contaminadas? Sí ~~No~~**

   ¿en qué forma? __________

5. **¿Tiene algún tipo de padecimiento que pueda asociar al agua contaminada?**

   Sí ~~No~~ ¿Cuál? __________

6. **¿Usted o alguien cercano a usted tiene cáncer?**

   Sí __________ **¿De qué tipo?** HEPATICO

   No __________ **¿Qué edad tiene?** __________

7. **¿En qué época considera más difícil la contaminación en el río?**

Encuesta Hábitos y salud

Nombre Adrián Hernández

Edad 55

1. **¿Fuma? Sí No** (No circled)
   Si su respuesta fue sí: ¿Con qué frecuencia de cigarrillos al día?

   — **cigarrillos al día.**

2. **¿Se encuentra tomando algún tratamiento médico? Sí No** (No circled)
   Si su respuesta fue sí, ¿de qué tipo?

   —

3. **¿Ingiere bebidas alcohólicas? Sí No** (No circled)
   Si su respuesta fue sí, ¿con qué frecuencia?

   — **días a la semana**

4. **¿Consume o está en contacto con las aguas contaminadas? Sí No** (No circled)
   ¿en qué forma? —

5. **¿Tiene algún tipo de padecimiento que pueda asociar al agua contaminada?**
   Sí No (No circled) **¿Cuál?** —

6. **¿Usted o alguien cercano a usted tiene cáncer?**
   Sí________ **¿De qué tipo?** —
   No (circled)________ **¿Qué edad tiene?**________

7. **¿En qué época considera más difícil la contaminación en el río?**
   Tiempo de sequías

Encuesta Hábitos y salud

Nombre Yennifer

Edad 24

1. **¿Fuma? Sí No** (No circled)
   Si su respuesta fue sí: ¿Con qué frecuencia de cigarrillos al día?

   — **cigarrillos al día.**

2. **¿Se encuentra tomando algún tratamiento médico? Sí No** (No circled)
   Si su respuesta fue sí, ¿de qué tipo?

   —

3. **¿Ingiere bebidas alcohólicas? Sí No** (No circled)
   Si su respuesta fue sí, ¿con qué frecuencia?

   — **días a la semana**

4. **¿Consume o está en contacto con las aguas contaminadas? Sí No** (No circled)
   ¿en qué forma? —

5. **¿Tiene algún tipo de padecimiento que pueda asociar al agua contaminada?**
   Sí No (No circled) **¿Cuál?** —

6. **¿Usted o alguien cercano a usted tiene cáncer?**
   Sí ________ **¿De qué tipo?** —
   No (circled) ________ **¿Qué edad tiene?** —

7. **¿En qué época considera más difícil la contaminación en el río?**
   Desde el 2023 hasta la fecha
   Sin observaciones

Encuesta Hábitos y salud

Nombre Patricia Gonzalez Sargoza

Edad 23

1. **¿Fuma?** (**Sí**) **No**
   Si su respuesta fue sí: ¿Con qué frecuencia de cigarrillos al día?

   2 **cigarrillos al día.**

2. **¿Se encuentra tomando algún tratamiento médico?** **Sí** (**No**)
   Si su respuesta fue sí, ¿de qué tipo?

   ____

3. **¿Ingiere bebidas alcohólicas?** (**Sí**) **No**
   Si su respuesta fue sí, ¿con qué frecuencia?

   1 **días a la semana**

4. **¿Consume o está en contacto con las aguas contaminadas?** **Sí** (**No**)
   ¿en qué forma? —

5. **¿Tiene algún tipo de padecimiento que pueda asociar al agua contaminada?**
   Sí (**No**) **¿Cuál?** —

6. **¿Usted o alguien cercano a usted tiene cáncer?**
   (**Sí**) ______ **¿De qué tipo?** Leucemia
   No ______ **¿Qué edad tiene?** 9 años

7. **¿En qué época considera más difícil la contaminación en el río?**
   En tiempo de lluvias

Encuesta Hábitos y salud

Nombre Isabel Flores

Edad 19

1. **¿Fuma?** **(Sí)** **No**
   Si su respuesta fue sí: ¿Con qué frecuencia de cigarrillos al día?

   7 **cigarrillos al día.**

2. **¿Se encuentra tomando algún tratamiento médico?** **Sí** **(No)**
   Si su respuesta fue sí, ¿de qué tipo?

   —

3. **¿Ingiere bebidas alcohólicas?** **(Sí)** **No**
   Si su respuesta fue sí, ¿con qué frecuencia?

   1 **días a la semana**

4. **¿Consume o está en contacto con las aguas contaminadas?** **Sí** **(No)**
   ¿en qué forma? —

5. **¿Tiene algún tipo de padecimiento que pueda asociar al agua contaminada?**
   Sí **(No)** **¿Cuál?** —

6. **¿Usted o alguien cercano a usted tiene cáncer?**
   Sí__________ **¿De qué tipo?** —
   (No)__________ **¿Qué edad tiene?** —

7. **¿En qué época considera más difícil la contaminación en el río?**
   Tiempo de lluvias

Encuesta Hábitos y salud

Nombre Alexa Gonzalez

Edad 17

1. **¿Fuma? Sí ~~No~~** (No circled)
   Si su respuesta fue sí: ¿Con qué frecuencia de cigarrillos al día?

   — **cigarrillos al día.**

2. **¿Se encuentra tomando algún tratamiento médico? Sí No** (No circled)
   Si su respuesta fue sí, ¿de qué tipo?

   —

3. **¿Ingiere bebidas alcohólicas? Sí No** (No circled)
   Si su respuesta fue sí, ¿con qué frecuencia?

   — **días a la semana**

4. **¿Consume o está en contacto con las aguas contaminadas? Sí No** (No circled)
   ¿en qué forma? —

5. **¿Tiene algún tipo de padecimiento que pueda asociar al agua contaminada?**
   Sí No (No circled) **¿Cuál?** —

6. **¿Usted o alguien cercano a usted tiene cáncer?**
   Sí________ **¿De qué tipo?** —
   No (circled)________ **¿Qué edad tiene?** —

7. **¿En qué época considera más difícil la contaminación en el río?**
   No lo considero

Encuestas

La Barca

✓

Consentimiento Informado

Nombre del proyecto en el cual habrá la participación:

Potencial genotóxico de las aguas de el Río Santiago en las poblaciones de El Salto, Juanacatlán, Cuenca del Ahogado, Puente Grande, La Barca y Jamay del estado de Jalisco y daño genético en linfocitos humanos de los habitantes vecinos.

Participantes voluntarios:

Habitantes de zonas aledañas al Río Santiago mayores de 18 años.

Información del proyecto en cual participan:

Práctica: donación de una gota de sangre obtenida por punción anular usando material nuevo y completamente limpio.

Información científica del proyecto a resolver.

Pregunta científica

Las aguas contaminadas del Río Santiago pueden producir mutaciones y cáncer?

Hipótesis:

Los componentes genotóxicos del agua de la cuenca Lerma-Chapala-Santiago poseen actividad genotóxica capaz de inducir daño genético en los habitantes de estas poblaciones detectable mediante la prueba del cometa.

Objetivo

Evaluar el potencial genotóxico de las aguas de el Río Santiago en El Salto del estado de Jalisco, en las poblaciones de La Barca y Jamay y el daño genético en linfocitos humanos de habitantes aledaños.

Decisión de participación

Descripción del proyecto. ¿Qué se va hacer?

Se donará una gota de sangre para evaluar el daño genético en células de sangre de personas expuestas a estas aguas.

Las actividades a realizar por parte de los individuos.

Donación de una gota de sangre periférica mediante punción anular.

Riesgo: No existe.

Manejo de datos. Usados de forma confidencial y para fines científicos.

Aceptación (sí) o **rechazo** (no) para participar.

Nombre	Aceptación Si ✓	No	Firma
Dolce M. Cruz V.	✓		[illegible]
Enya Mechelle Diaz M.	✓		Enya
Yudith Garcia	✓		[illegible]
Marcela Gutierrez	✓		Marcela Gutierrez
Sergio Moreno	✓		Sergio Moreno
José Moreno	✓		José Moreno
veronica Hernandes	✓		veronica Hernandes
Juan Ramon P.	✓		[illegible]
Mª Sanchez A.	—		[illegible]
Silvia De la Cruz	✓		[illegible]

Número telefónico para información relacionada con los resultados: 37771191 ext 33207 Doctor Carlos Álvarez moya

Encuesta Hábitos y salud

Nombre Silvia de la Cruz.

Edad 32

1. **¿Fuma? Sí ~~No~~**

   Si su respuesta fue sí: ¿Con qué frecuencia de cigarrillos al día?

   ___________ **cigarrillos al día.**

2. **¿Se encuentra tomando algún tratamiento médico? Sí ~~No~~**

   Si su respuesta fue sí, ¿de qué tipo?

   ____

3. **¿Ingiere bebidas alcohólicas? Sí ~~No~~**

   Si su respuesta fue sí, ¿con qué frecuencia?

   ___________ **días a la semana**

4. **¿Consume o está en contacto con las aguas contaminadas? Sí ~~No~~**

   ¿en qué forma? ___________________________________

5. **¿Tiene algún tipo de padecimiento que pueda asociar al agua contaminada?**

   ~~Sí~~ No ¿Cuál? ___________________________________

6. **¿Usted o alguien cercano a usted tiene cáncer?**

   ~~Sí~~ ___________________ **¿De qué tipo?** Páncreas

   No ___________________ **¿Qué edad tiene?** ___________________

7. **¿En qué época considera más difícil la contaminación en el río?**

Encuesta Hábitos y salud

Nombre Enya Michel Diaz Martinez

Edad 24

1. **¿Fuma? Sí No** (circled: No)
   Si su respuesta fue sí: ¿Con qué frecuencia de cigarrillos al día?

   — **cigarrillos al día.**

2. **¿Se encuentra tomando algún tratamiento médico? Sí No** (circled: No)
   Si su respuesta fue sí, ¿de qué tipo?

   —

3. **¿Ingiere bebidas alcohólicas? Sí No** (circled: No)
   Si su respuesta fue sí, ¿con qué frecuencia?

   — **días a la semana**

4. **¿Consume o está en contacto con las aguas contaminadas? Sí No** (circled: No)
   ¿en qué forma?

5. **¿Tiene algún tipo de padecimiento que pueda asociar al agua contaminada?**
   Sí No (circled: Sí) **¿Cuál?** Alergias

6. **¿Usted o alguien cercano a usted tiene cáncer?**
   Sí ______ **¿De qué tipo?** —
   No (circled) ______ **¿Qué edad tiene?**

7. **¿En qué época considera más difícil la contaminación en el río?**
   En la actualidad todo el año

Encuesta Hábitos y salud

Nombre SERGIO MORENO

Edad 50

1. ¿Fuma? Sí No

   Si su respuesta fue sí: ¿Con qué frecuencia de cigarrillos al día?

   20 cigarrillos al día.

2. ¿Se encuentra tomando algún tratamiento médico? Sí No

   Si su respuesta fue sí, ¿de qué tipo?

   ____

3. ¿Ingiere bebidas alcohólicas? Sí No

   Si su respuesta fue sí, ¿con qué frecuencia?

   ________ días a la semana

4. ¿Consume o está en contacto con las aguas contaminadas? Sí No

   ¿en qué forma? ______________________________

5. ¿Tiene algún tipo de padecimiento que pueda asociar al agua contaminada?

   Sí No ¿Cuál? ______________________________

6. ¿Usted o alguien cercano a usted tiene cáncer?

   Sí ______________ ¿De qué tipo? ______________

   No ______________ ¿Qué edad tiene? ______________

7. ¿En qué época considera más difícil la contaminación en el río?

VERANO, MEDIA.

Encuesta Hábitos y salud

Nombre Veronica Hernandez

Edad 47

1. ¿Fuma? Sí (No)

   Si su respuesta fue sí: ¿Con qué frecuencia de cigarrillos al día?

   ________cigarrillos al día.

2. ¿Se encuentra tomando algún tratamiento médico? (Sí) No

   Si su respuesta fue sí, ¿de qué tipo?

   Migraña

3. ¿Ingiere bebidas alcohólicas? Sí (No)

   Si su respuesta fue sí, ¿con qué frecuencia?

   ________días a la semana

4. ¿Consume o está en contacto con las aguas contaminadas? (Sí) No

   ¿en qué forma? Ejercicio cada 3er dya

5. ¿Tiene algún tipo de padecimiento que pueda asociar al agua contaminada?

   (Sí) No ¿Cuál? Migraña.

6. ¿Usted o alguien cercano a usted tiene cáncer?

   Sí________ ¿De qué tipo?________

   (No)________ ¿Qué edad tiene?________

7. ¿En qué época considera más difícil la contaminación en el río?

   Lluvia.

Encuesta Hábitos y salud

Nombre Judit Garcia

Edad 30

1. ¿Fuma? Sí (No)
   Si su respuesta fue sí: ¿Con qué frecuencia de cigarrillos al día?

   — cigarrillos al día.

2. ¿Se encuentra tomando algún tratamiento médico? Sí (No)
   Si su respuesta fue sí, ¿de qué tipo?

   —

3. ¿Ingiere bebidas alcohólicas? Sí (No)
   Si su respuesta fue sí, ¿con qué frecuencia?

   — días a la semana

4. ¿Consume o está en contacto con las aguas contaminadas? Sí (No)
   ¿en qué forma? —

5. ¿Tiene algún tipo de padecimiento que pueda asociar al agua contaminada?
   Sí (No) ¿Cuál? —

6. ¿Usted o alguien cercano a usted tiene cáncer?
   Sí ________ ¿De qué tipo? —
   (No) ________ ¿Qué edad tiene? —

7. ¿En qué época considera más difícil la contaminación en el río?
   Todo el año

Encuesta Hábitos y salud

Nombre Dulce María Cruz V

Edad 17

1. **¿Fuma? Sí No** (circled: No)
   Si su respuesta fue sí: ¿Con qué frecuencia de cigarrillos al día?

   ______ **cigarrillos al día.**

2. **¿Se encuentra tomando algún tratamiento médico? Sí No** (circled: No)
   Si su respuesta fue sí, ¿de qué tipo?

   ______

3. **¿Ingiere bebidas alcohólicas? Sí No** (circled: No)
   Si su respuesta fue sí, ¿con qué frecuencia?

   ______ **días a la semana**

4. **¿Consume o está en contacto con las aguas contaminadas? Sí No** (circled: No)
   ¿en qué forma? ______

5. **¿Tiene algún tipo de padecimiento que pueda asociar al agua contaminada?**
   Sí No ¿Cuál? Enfermedades en los pulmones

6. **¿Usted o alguien cercano a usted tiene cáncer?**
   Sí ______ **¿De qué tipo?** ______
   No (circled) ______ **¿Qué edad tiene?** ______

7. **¿En qué época considera más difícil la contaminación en el río?**
   Cuando tiran mas basura puede ser

Encuesta Hábitos y salud

Nombre Ma. Sánchez Ávila

Edad 52

1. **¿Fuma? Sí (No)**
   Si su respuesta fue sí: ¿Con qué frecuencia de cigarrillos al día?

   — **cigarrillos al día.**

2. **¿Se encuentra tomando algún tratamiento médico? Sí (No)**
   Si su respuesta fue sí, ¿de qué tipo?

   —

3. **¿Ingiere bebidas alcohólicas? Sí (No)**
   Si su respuesta fue sí, ¿con qué frecuencia?

   — **días a la semana**

4. **¿Consume o está en contacto con las aguas contaminadas? (Sí) No**
   ¿en qué forma? Exposición directa.

5. **¿Tiene algún tipo de padecimiento que pueda asociar al agua contaminada?**
   Sí (No) ¿Cuál? —

6. **¿Usted o alguien cercano a usted tiene cáncer?**
   (Sí) ¿De qué tipo? de Páncreas
   No ¿Qué edad tiene? 70

7. **¿En qué época considera más difícil la contaminación en el río?**
   En tiempo de lluvias.

Observación:
- Ve muy sucio el lago de Chapala.
- Presencia de mosco
- Asocia otras enfermedades como la Diabetes
- Presencia de Dengue
- Fumigan con muy poca regularidad.
- Mosquero en Ocotlán

Encuesta Hábitos y salud

Nombre Jose Moreno

Edad 73

1. **¿Fuma? Sí (No)**

   Si su respuesta fue sí: ¿Con qué frecuencia de cigarrillos al día?

   ______ **cigarrillos al día.**

2. **¿Se encuentra tomando algún tratamiento médico? (Sí) No**

   Si su respuesta fue sí, ¿de qué tipo?

   Hipertensión

3. **¿Ingiere bebidas alcohólicas? Sí (No)**

   Si su respuesta fue sí, ¿con qué frecuencia?

   ______ **días a la semana**

4. **¿Consume o está en contacto con las aguas contaminadas? Sí No**

   ¿en qué forma? No, cruzando

5. **¿Tiene algún tipo de padecimiento que pueda asociar al agua contaminada?**

   Sí No ¿Cuál? ______

6. **¿Usted o alguien cercano a usted tiene cáncer?**

   Sí ______ **¿De qué tipo?** ______

   No — ______ **¿Qué edad tiene?** ______

7. **¿En qué época considera más difícil la contaminación en el río?**

   Brisa mucho cuando esta lloviendo, en la cienega se cultiva maiz, el agua no esta muy sucia, pesca (tilapia), sucio para bañarse, riegan maiz con el agua embalsada.

Encuesta Hábitos y salud

Nombre Juan Ramón Pacheco

Edad 32

1. **¿Fuma? (Sí) No**

   Si su respuesta fue sí: ¿Con qué frecuencia de cigarrillos al día?

   7 **cigarrillos al día.**

2. **¿Se encuentra tomando algún tratamiento médico? Sí (No)**

   Si su respuesta fue sí, ¿de qué tipo?

   ____

3. **¿Ingiere bebidas alcohólicas? Sí (No)**

   Si su respuesta fue sí, ¿con qué frecuencia?

   ________ **días a la semana**

4. **¿Consume o está en contacto con las aguas contaminadas? Sí No**

   ¿en qué forma? Turismo, paso

5. **¿Tiene algún tipo de padecimiento que pueda asociar al agua contaminada?**

   Sí (No) ¿Cuál? ______________________

6. **¿Usted o alguien cercano a usted tiene cáncer?**

   Sí ______________ **¿De qué tipo?** ______________

   No — ______________ **¿Qué edad tiene?** ______________

7. **¿En qué época considera más difícil la contaminación en el río?**

   Olor Fétido, conexión con el río Santiago en Ocotlan, hace 18 años era más limpia, 19 años antes pasaba, agua revolcada, se seca en el río totalmente a veces

Encuesta Hábitos y salud

Nombre Marcela Gutierrez

Edad 70

1. **¿Fuma? Sí No** (circled: No)
   Si su respuesta fue sí: ¿Con qué frecuencia de cigarrillos al día?

   ______ **cigarrillos al día.**

2. **¿Se encuentra tomando algún tratamiento médico? Sí No** (circled: Sí)
   Si su respuesta fue sí, ¿de qué tipo?

   Corazón, depresión

3. **¿Ingiere bebidas alcohólicas? Sí No** (circled: No)
   Si su respuesta fue sí, ¿con qué frecuencia?

   ______ **días a la semana**

4. **¿Consume o está en contacto con las aguas contaminadas? Sí No** (circled: Sí)
   ¿en qué forma? Solo vive cerca

5. **¿Tiene algún tipo de padecimiento que pueda asociar al agua contaminada?**
   Sí No (circled: No) **¿Cuál?** ______

6. **¿Usted o alguien cercano a usted tiene cáncer?**
   Sí ______ **¿De qué tipo?** ______
   No (circled) ______ **¿Qué edad tiene?** ______

7. **¿En qué época considera más difícil la contaminación en el río?**
   Cuando llueve.
